# CTHRC1 Attenuates Tendinopathy via Enhancing EGFR/MAPK Signaling Pathway

**DOI:** 10.1002/advs.202406611

**Published:** 2024-11-14

**Authors:** Cheng Chen, Xu Zheng, Cheng Wang, HaiChao Zhou, Yi Zhang, TianBao Ye, YunFeng Yang

**Affiliations:** ^1^ Department of Orthopedics Tongji Hospital School of Medicine Tongji University Shanghai 200065 China; ^2^ Department of Bone and Joint Surgery Department of Orthopedics Renji Hospital School of Medicine Shanghai 200001 China; ^3^ Department of Orthopedics Shanghai Sixth People's Hospital Affiliated to Shanghai Jiao Tong University School of Medicine Shanghai 200233 China; ^4^ Xiamen Cardiovascular Hospital of Xiamen University School of Medicine Xiamen University Xiamen Fujian 361008 China; ^5^ Shanghai Sixth People’s Hospital Affiliated to Shanghai Jiao Tong University School of Medicine Shanghai 200233 China; ^6^ School of Medicine Tongji University Shanghai 200092 China; ^7^ Department of Orthopaedics Ruijin Hospital Shanghai Jiao Tong University School of Medicine Shanghai 200025 China

**Keywords:** CTHRC1, EGFR, MAPK, tendinopathy, tendon stem/progenitor cell

## Abstract

Tendinopathy poses a formidable challenge due to the inherent limitations of tendon regenerative capabilities post‐injury. At present, effective curative approaches for tendinopathy are still lacking. Collagen triple helix repeat‐containing 1 (CTHRC1) is an extracellular matrix protein with significant roles in both physiological and pathological processes. The present study aims to investigate the function and underlying mechanism of CTHRC1 in tendinopathy. In this study, CTHRC1 is identified as a potential effector in promoting tendon regeneration through multi‐proteomic analysis of Achilles tendon tissues in mice. In vitro, CTHRC1 enhances the proliferation, migration, and tenogenic differentiation of tendon stem/progenitor cell (TSPC). In vivo, CTHRC1 deletion impairs tendon healing, while its overexpression reverses the detrimental effects caused by CTHRC1 deficiency. Mechanistically, proteomics on TSPC stimulated with recombinant CTHRC1 reveal that CTHRC1 activates the mitogen‐activated protein kinase (MAPK) signaling pathway via binding to epidermal growth factor receptor (EGFR), which in turn promotes the proliferative, migrative, and tenogenic capacities of TSPC to attenuate Achilles tendinopathy. Conversely, inhibiting EGFR reverses the tendon‐healing effect of CRHRC1. The study demonstrates that CTHRC1 can promote the proliferative, migrative, and tenogenic capacities of TSPC, ultimately facilitating tendon healing through activating the EGFR/MAPK signaling pathway. CTHRC1 holds promise as a potential intervention for tendinopathy.

## Graphical abstract

Tendinopathy poses a formidable challenge due to the inherent limitations of tendon regenerative capabilities post‐injury. In this study, CTHRC1 is identified as a potential effector in promoting tendon regeneration through multi‐proteomic analysis of Achilles tendon tissues in mice. The study demonstrates that CTHRC1 can promote the proliferative, migrative, and tenogenic capacities of TSPC, ultimately facilitating tendon healing through activating the EGFR/MAPK signaling pathway.

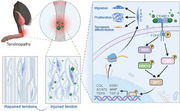

## 1 Introduction

The tendon, a dense connective tissue, exhibits a highly organized structure comprised predominantly of collagen fibers with a sparse distribution of cells, primarily tenocytes. This highly arranged structure of collagen‐riched extracellular matrix endows tendons with exceptional strength, facilitating the transmission of tremendous forces between muscles and bones. Tendinopathy is a prevalent tendon disorder,^[^ ^1^, ^2^ ^]^ characterized by abnormal cell infiltration, disrupted extracellular matrix remodeling, and disordered collagen arrangement. These changes culminate in a decline in tendon biomechanical properties and elicit clinical symptoms, including pain, swelling, and reduced functional performance.^[^ ^3^ ^]^ The pathogenesis of tendinopathy is multifactorial and complex, while extracellular matrix remodeling and maladaptive repair play a crucial role. The challenge in treating tendinopathy can be partly attributed to the reduced capacity for tendon regeneration following injury in adults.^[^ ^4^, ^5^, ^6^ ^]^ Currently, much research focuses on the mechanisms and treatment approaches for tendinopathy,^[^ ^7^, ^8^, ^9^, ^10^, ^11^ ^]^ but effective therapies are still lacking.

Studies have revealed a significant regenerative capacity for tendon in newborn.^[^ ^5^, ^6^, ^12^ ^]^ Transitioning from a fetal‐like reparative state to a functional tendon maturity is crucial for tendon regeneration. Tendon stem/progenitor cell (TSPC) holds the key to this process.^[^ ^13^, ^14^, ^15^ ^]^ TSPC is a type of multipotent stem cell present in tendon tissue, possessing self‐renewal and multilineage differentiation capabilities.^[^ ^15^ ^]^ Compared to other stem cell types, TSPC exhibits a more pronounced expression of tendon marker genes and demonstrates enhanced potential in promoting tendon healing, reflecting its superior capacity for tendon regeneration.^[^ ^14^, ^16^, ^17^, ^18^ ^]^ Ensuring the sufficient amount and functionality of TSPC are pivotal for tendon regeneration after injury. Hence, promoting migration, proliferation, and tenogenic differentiation of TSPC can facilitate tendon regeneration.^[^ ^19^, ^20^, ^21^ ^]^ However, the current strategies for regulating TSPC mobilizing to the injured area, facilitating TSPC proliferation, and enhancing their tenogenic differentiation for tendon regeneration remain very limited.

Collagen triple helix repeat containing 1 (CTHRC1) is a secreted extracellular matrix glycoprotein and originally identified in balloon‐injured arteries.^[^ ^22^ ^]^ It is widely reported to extensively participate in a variety of physiological and pathological processes.^[^ ^23^ ^]^ Previous studies have demonstrated the significant role of CTHRC1 in the repair of various injuries, such as vascular remodeling,^[^ ^22^, ^24^ ^]^ post‐myocardial infarction repair,^[^ ^25^, ^26^ ^]^ and skin wound healing.^[^ ^27^, ^28^ ^]^ However, the potential role and underlying mechanism of CTHRC1 in tendinopathy remain unknown.

In this study, we identified CTHRC1 as a potential effector in promoting tendon regeneration through multi‐proteomics analysis in mice. We demonstrated that CTHRC1 could promote tendon healing and attenuate tendinopathy by enhancing the proliferation, migration, and tenogenic differentiation of TSPC post‐injury. In contrast, CTHRC1 deletion exacerbated tendinopathy in vivo. Mechanistically, we found that CTHRC1 could activate the mitogen‐activated protein kinase (MAPK) signaling pathway via binding to epidermal growth factor receptor (EGFR) in TSPC, further promoting the proliferative, migrative, and tenogenic differentiative capacities of TSPC. Our study highlights the positive role of CTHRC1 in tendon regeneration and suggests that CTHRC1 may be a promising new option for tendinopathy treatment.

## 2 Results

### 2.1 CTHRC1 Plays a Critical Role in Achilles Tendon Regeneration and Healing

**Figure 1:**
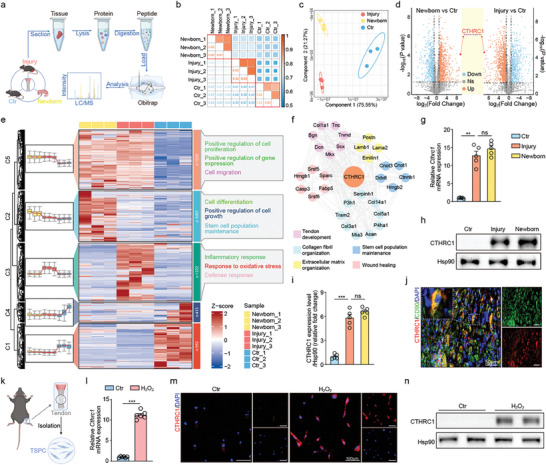
CTHRC1 plays a critical role in tendon regeneration and healing. a) Schematic overview of proteomics on Achilles tendon tissues from newborn mice, adult mice with Achilles tendinopathy, and normal adult mice. b) Inter‐sample Spearman correlation heat map, with numbers representing correlation coefficient. c) PLS‐DA plot of the proteomic data. d) Volcano plot showing CTHRC1 markedly up‐regulated in both newborn and injured mice's Achilles tendon. e) Heatmap of protein expression profiles with GO biological process annotation of clusters. f) CTHRC1‐centered protein–protein interaction network. g) mRNA expression level of CTHRC1 in Achilles tendon in different mice groups (n = 5). h) Representative western blot of CTHRC1 in Achilles tendon in different mice groups. i) Quantification of western blot of CTHRC1 in Achilles tendon in different mice groups (n = 5). j) Colocalization of CTHRC1 and CD90 in tendon tissue by immunofluorescence. k) Schematic of the experimental protocol of TSPC isolation. l) CTHRC1 mRNA expression level in different TSPC groups (n = 6). m) Representative immunofluorescent image of TSPC stained with CTHRC1 antibody. n) Representative western blot of CTHRC1 in different TSPC groups. Data are presented as mean ± SEM. g and i, by one‐way ANOVA; l, by unpaired t‐test. ^**^ *P* < 0.01; ^***^ *P* < 0.001; ns, no significance. TSPC: tendon stem/progenitor cell.

Given that newborn mice possess a remarkable capacity for tendon healing after injury compared with adult mice,^[^ ^5^, ^12^ ^]^ analyzing the protein expression profiles of newborn and adult mice can help identify key factors that promote the regeneration process.^[^ ^4^ ^]^ Tendinopathy often arises as a consequence of maladaptive tendon healing post‐injury, and Achilles tendon injury is widely used as a tendinopathy model.^[^ ^7^ ^]^ Therefore, we conducted proteomics on Achilles tendon tissues derived from newborn mice (Newborn), adult mice (Ctr), and adult mice with Achilles tendon injury (Injury), to systematically analyze the alteration of protein expression profiles after tendon maturation and injury (**Figure** **1**a). The correlation heatmap showed strong positive intra‐group correlations, while the inter‐group correlations were less prominent (Figure 1b). Similarly, the partial least squares‐discriminant analysis (PLS‐DA) revealed a clear separation between groups and proximity within groups (Figure 1c). These results showed the consistency of protein expression profiles within the sample groups and the difference in protein expression profiles among the groups.

The volcano plot displayed that there existed many differently expressed proteins (DEPs) between tendons in newborn and adult mice, as well as between normal tendons and injured tendons, among which CTHRC1 was significantly elevated in both adult mice with Achilles tendon injury and newborn mice (Figure 1d). The heatmap illustrated that the newborn mice possessed more active biological processes of stem cell maintenance, cell differentiation, and cell growth, while these processes diminished significantly in adult mice (Figure 1e). Besides, tendon injury led to obvious inflammatory response and oxidative stress, associated with tissue damage. Consistent with previous results, Gene Set Enrichment Analysis (GSEA) analysis also revealed that newborn mice tendons had active biological processes of stem cell maintenance, differentiation, cell proliferation, and collagen fiber organization, associated with higher tendon healing ability (Figure S1, Supporting Information). On the other side, enhanced immune response and extracellular matrix disassembly were enriched in injured tendon, associated with inflammatory cell infiltration and poor repair process (Figure S2, Supporting Information).

Next, we identified the intersection of DEPs from the two comparisons (Newborn vs Ctr, Injury vs Ctr) to verify the potential protective factors. Gene Ontology (GO) analysis revealed that these DEPs were enriched in the cytoplasm, nucleus, and membrane, with the functions of structural constituent of the ribosome, nucleotide binding, and cadherin binding involved in cell–cell adhesion (Figure S3a,b, Supporting Information). Protein‐protein interaction (PPI) network analysis revealed that there existed a high degree of connectivity among CTHRC1 and DEP clusters related to collagen fibril organization, extracellular matrix organization, wound healing, tendon development, and stem cell population maintenance, which play an essential part during repair response upon injury (Figure 1f). Given this, we initially proposed a hypothesis that CTHRC1 would act as an important factor in promoting tendon regeneration after injury. Besides, PPI using the proteomic data from the in vivo injury model demonstrated the interactions between EGFR/MAPK pathway and collagen fibril organization, tendon development, regeneration, wound healing, and stem cell proliferation and development (Figure S3c, Supporting Information).

To further confirm the expression pattern of CTHRC1 in Achilles tendon, we conducted both quantitative real‐time polymerase chain reaction (qRT‐PCR) analysis and western blot. The results revealed that CTHRC1 was significantly upregulated in adult mice with Achilles tendon injury and newborn mice, compared to normal adult mice, consistent with the previous findings (Figure 1g–i). Additionally, we further investigated the temporal expression pattern of CTHRC1 in adult mice with Achilles tendon injury and found that CTHRC1 level rose transiently after tendon injury, but consistently fell over the next few weeks (Figure S4a‐c, Supporting Information).

We performed experiments on TSPC, endothelial cells, and immunocytes, which serve as key effector cells in the damage response and regulation of the repair process after tendon injury.^[^ ^20^, ^29^, ^30^, ^31^, ^32^ ^]^ To explore the possible source of CTHRC1 in injured tendon, immunofluorescence was used to detect the co‐localization of CTHRC1 with CD90 (as the marker for TSPC), CD45 (as the marker for immunocytes), and CD31 (as the marker for endothelial cells) in tendon tissue after injury. The immunofluorescence assay showed that CTHRC1 co‐localized most with CD90 (Figure 1j), but not with CD45 and CD31 (Figure S5a,b, Supporting Information). This indicates that TSPC is the main source of CTHRC1, rather than immunocytes or endothelial cells. In vitro, according to previous research,^[^ ^19^ ^]^ we isolated TSPC (Figures 1k and S6, Supporting Information). To mimic the injury status in vitro, we stimulated these cells with H_2_O_2_ as previously reported.^[^ ^19^ ^]^ qRT‐PCR, immunofluorescence, and western blot assays showed that CTHRC1 expression increased in H_2_O_2_‐stimulated TSPC (Figures 1l–n and S7a,b, Supporting Information). However, no significant change in CTHRC1 expression was observed in endothelial cells (C166) and macrophages (Raw264.7) stimulated with H_2_O_2_ (Figure S8a,b, Supporting Information). Based on these results, we speculated that TSPC should be the primary source of CTHRC1 after tendon injury.

### 2.2 CTHRC1 Promotes the Proliferation, Migration, and Tenogenic Differentiation of TSPC In Vitro

TSPC is essential for efficient tendon healing after injury. The proliferation, migration to the injured site, and tenogenic differentiation of TSPC are important processes for tendon regeneration after injury.^[^ ^19^, ^20^, ^21^ ^]^ Given that the previous proteomic analysis in Figure 1 revealed a high correlation of CTHRC1 with stem cell maintenance and proliferation, tendon development, and collagen fiber biosynthesis and organization. Therefore, we investigated the effects of CTHRC1 on TSPC. We performed cell counting kit‐8 assay (CCK‐8) and lactate dehydrogenase (LDH) cytotoxicity assay to identify the optimal concentration of recombined CTHRC1 (rCTHRC1) for stimulation (Figure S9a,b, Supporting Information). The CCK‐8 assay showed that as the concentration increased from 0 to 1000 ng mL^−1^, cell proliferation was also enhanced. However, there was no significant difference between the concentrations of 1000 and 2000 ng mL^−1^ (Figure S9a, Supporting Information). On the other hand, the LDH assay showed no significant difference in cell toxicity at concentrations from 0 to 1000 ng mL^−1^, but cell toxicity was observed at a concentration of 2000 ng mL^−1^ (Figure S9b, Supporting Information). As a result, the optimal concentration of rCTHRC1 for stimulating TSPC was identified as 1 µg mL^−1^.

**Figure 2:**
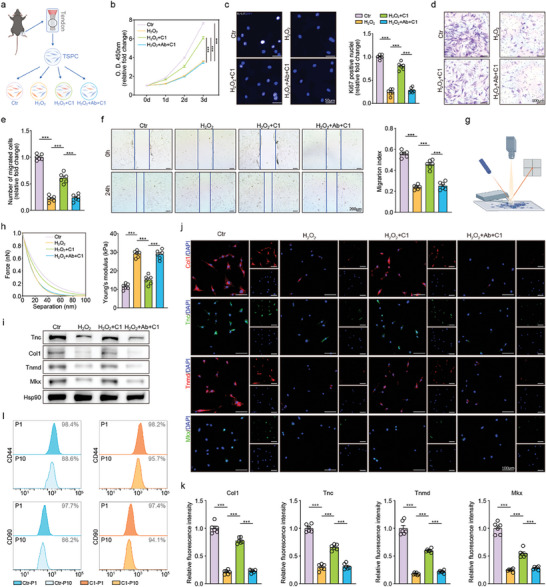
CTHRC1 promotes TSPC proliferation, migration, and tenogenic potentials in vitro. a) Schematic of the experimental protocol. b) TSPC proliferation curves at indicated time (n = 6). c) Representative immunofluorescent images of proliferative TSPC with Ki67‐labeled nuclei and quantification (n = 6). d) Representative images of transwell assay at 24 h. e) Quantification of transwell assay (n = 6). f) Representative images of wound healing assay and quantification (n = 6). g) Schematic of AFM testing. h) Representative AFM curves of TSPC and quantification of Young's modulus by AFM analysis (n = 6). i) Representative western blot images of indicated proteins in TSPC. j) Representative immunofluorescent micrographs of indicated proteins in TSPC. k) Immunofluorescence quantification of indicated proteins in TSPC (n = 6). l) Representative flow cytometric analysis of TSPC with passages. Data are presented as mean ± SEM. b, by two‐way ANOVA; c, e, f, h, and k, by one‐way ANOVA. ^***^ *P* < 0.001. TSPC: tendon stem/progenitor cell; C1: CTHRC1; Ab: anti‐CTHRC1 antibody; P: passage.

Subsequently, administration of rCTHRC1 was employed to investigate the effects of CTHRC1 on TSPC during injury in vitro (**Figure** **2**a). Both the CCK‐8 and Ki67 immunofluorescence assays revealed that the proliferation of TSPC was inhibited upon H_2_O_2_ stimulation, while administration with rCTHRC1 rescued this phenomenon, indicating that CTHRC1 could significantly enhance the proliferation of TSPC during injury (Figure 2b,c).

Besides, the transwell and wound healing assays showed that the inhibition of TSPC migration after H_2_O_2_ stimulation was reversed by rCTHRC1, showing that CTHRC1 could significantly increase the migration ability of TSPC (Figure 2d–f). The atomic force microscopy (AFM) experiments revealed that the higher microscopic Young's modulus of TSPC induced by H_2_O_2_ stimulation was restored under treatment with rCTHRC1 (Figure 2g,h). The increase in cellular microscopic Young's modulus is associated with cellular senescence and decreased cell motility.^[^ ^33^, ^34^, ^35^ ^]^ These findings indicated that rCTHRC1 could enhance the migration capability of TSPC.

Moreover, H_2_O_2_ could induce the inhibition of tenogenic marker mRNA expression in TSPCs, while the administration with rCTHRC1 upregulated these tenogenic marker mRNA expressions. Further cotreatment with CTHRC1 antibody, in turn, downregulated the expression again, indicating that rCTHRC1 significantly enhanced the tenogenic capability of TSPC (Figure S10, Supporting Information). Similar trends were observed by western blot and immunofluorescence assays (Figures 2i–k and S11, Supporting Information). Additionally, this effect of CTHRC1 on promoting tenogenic capacity became more pronounced as the concentration increased (Figure S12a,b, Supporting Information). To sum up, these results hinted that CTHRC1 could enhance the proliferation, migration, and tenogenic differentiation of TSPC, which were crucial for the healing response of the injured tendon.

Stem cells are intrinsically endowed with a potent regenerative capacity for tissue regeneration, which is of paramount importance in the repair of damaged tissues. A decline in stem cell functionality caused by the deficiency of stem cell maintenance significantly impairs this regenerative process.^[^ ^36^, ^37^ ^]^ To preliminary verify the effect of CTHRC1 on stem cell population maintenance, we applied the CD44 and CD90 as stemness indicators of TSPC in flow cytometry. In the rCTHRC1‐stimulated group, the proportion of CD44 and CD90‐positive cells at the tenth passage was significantly higher than that in the control group (Figures 2l and S13a, Supporting Information). In addition, the qRT‐PCR and western blot results indicated that the expression of Oct4 and Sox2, serving as stemness indicators, in the rCTHRC1‐stimulated TSPC was higher at the tenth passage compared to the control (Figure S13b,c, Supporting Information). These results collectively suggested that CTHRC1 contributed to maintaining the stemness of TSPC. Hence, CTHRC1 may be beneficial to tendon repair after injury.

### 2.3 CTHRC1‐Deletion Worsens Tendon Regeneration Capacity After Injury

Given the positive effects of CTHRC1 on TSPC and the crucial role of TSPC in tendon regeneration, we further investigated the role of CTHRC1 in tendon injury in vivo induced by collagenase injection. First, we verified the successful generation of CTHRC1 knockout (KO) mice via western blot (Figure S14, Supporting Information). There was no significant difference between WT mice and CTHRC1 KO mice without tendon injury, according to the histopathological, biomechanical, and molecular analysis of the Achilles tendon (Figure S15a‐e, Supporting Information). This indicated that CTHRC1‐deletion has no effect on the tendon of normal mice.

**Figure 3:**
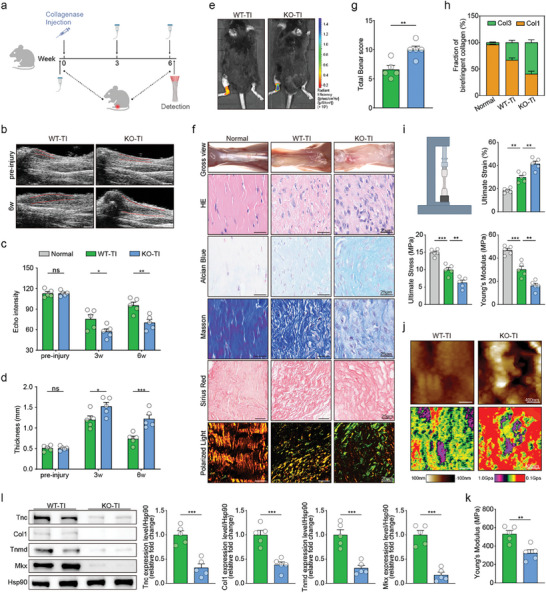
CTHRC1 knockout (KO) worsens tendon repair. a) Diagram of the experimental approach. b) Representative ultrasound imaging of Achilles tendon. c) Assessment of echo intensity at different time points (n = 5). d) Assessment of Achilles tendon thickness at different time points (n = 5). e) Representative in vivo fluorescence imaging. f) Representative images of gross view, hematoxylin & eosin (HE), Alcian Blue, Masson, Sirius Red staining, and Sirius Red staining using polarized light microscopy. g) Assessment of Achilles tendon using total Bonar score (n = 5). h) Fraction of birefringent collagen in Sirius Red staining imaged under polarized light (n = 5). i) Schematic of Achilles tendon mechanical testing and quantification of ultimate strain, ultimate stress, and Young's modulus (n = 5). j) Representative atomic force microscopy (AFM) images of Achilles tendon. k) Assessment of Young's modulus by AFM analysis (n = 5). l) Representative western blot images of indicated proteins in mice's Achilles tendon and quantification (n = 5). Data are presented as mean ± SEM. c and d, by two‐way ANOVA; g, k, and l, by unpaired t‐test; i, by one‐way ANOVA. ^*^ *P* < 0.05; ^**^ *P* < 0.01; ^***^ *P* < 0.001; ns, no significance. WT: wild‐type; KO: CTHRC1 knockout; TI: tendon injury.

Then, we performed a series of in vivo experiments to verify whether CTHRC1‐deletion could deteriorate the healing process of the Achilles tendon (**Figure** **3**a). A temporal ultrasound examination was used to assess the thickness and echo intensity of the Achilles tendon with the disease progression. Increased tendon thickness and decreased echo intensity are indicators of poorer tendon tissue quality and poorer repair.^[^ ^38^ ^]^ There were no differences in Achilles tendon thickness and echo intensity between the KO and WT mice before modeling (Figure 3b–d). However, at week 3 and 6 post‐injury, the tendons of KO mice were thicker and had lower echo intensity compared to WT mice, indicating poorer tendon repair (Figure 3b–d). Additionally, compared to WT mice, in vivo imaging detected significantly enhanced matrix metalloproteinases (MMP) activity in the KO mice (Figures 3e and S16a, Supporting Information). The heightened MMP activity suggests enhanced degradation of the extracellular matrix after tendon injury, associated with unfavorable tendon repair outcomes.^[^ ^39^, ^40^, ^41^ ^]^

The Bonar score was used to evaluate the extent of tendinopathy, with higher scores indicating more severe tendinopathy.^[^ ^7^ ^]^ Compared to WT mice, the gross morphologic and histological analysis of the Achilles tendon showed more pronounced inflammatory cell infiltration, ground substance accumulation, and collagen fiber disorganization in the KO mice, presenting with higher total Bonar scores (Figure 3f,g). Besides, as collagen type I is the main collagen component in the tendon under normal circumstances,^[^ ^32^, ^42^ ^]^ an abnormal increase in collagen type III can lead to biomechanical degradation of the tendon, signifying poorer tendon repair.^[^ ^43^ ^]^ Our result showed that CTHRC1 KO mice increased collagen type III percent, indicating poorer tendon healing (Figure 3f,h).

Additionally, mechanical testing serves as a valuable means to assess the key characteristics of tendon function. There was a significantly weaker macroscopic mechanical strength in the Achilles tendons of KO mice, characterized by elevated ultimate strain, reduced ultimate stress, and decreased Young's modulus (Figure 3i). To further assess the microscopic mechanical properties of the Achilles tendon, the detection of microscopic Young's modulus was performed using AFM. Consistently, AFM showed lower microscopic Young's modulus in the Achilles tendons of KO mice, indicating a worse ultrastructural repair^[^ ^19^ ^]^ (Figure 3j,k). Furthermore, the expressions of tenogenic genes were significantly downregulated in the Achilles tendons of KO mice, indicating a less efficient healing process (Figures 3l and S16b, Supporting Information).

Generally, these results demonstrated that CTHRC1 KO deteriorates tendon repair capacity after injury in mice.

### 2.4 Overexpressing CTHRC1 Improves Tendon Repair Ability

**Figure 4:**
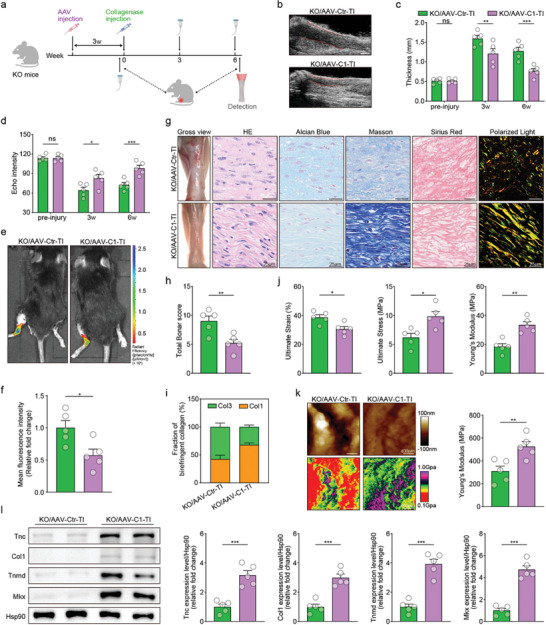
Adeno‐associated virus overexpressing CTHRC1 (AAV‐C1) improves tendon repair in KO mice. a) Diagram of the experimental approach. b) Representative ultrasound imaging of Achilles tendon at week 6 post‐injury. c) Assessment of Achilles tendon thickness at different time points (n = 5). d) Assessment of echo intensity at different time points (n = 5). e) Representative in vivo fluorescence imaging. f) Quantification of mean fluorescence intensity (n = 5). g) Representative images of gross view, HE, Alcian Blue, Masson, Sirius Red staining, and Sirius Red staining using polarized light microscopy. h) Assessment of Achilles tendon using total Bonar score (n = 5). i) Fraction of birefringent collagen in Sirius Red staining imaged under polarized light (n = 5). j) Assessment of ultimate strain, ultimate stress, and Young's modulus of Achilles tendon (n = 5). k) Representative AFM images of Achilles tendon and assessment of Young's modulus by AFM analysis (n = 5). l) Representative western blot images of indicated proteins in Achilles tendon and quantification (n = 5). Data are presented as mean ± SEM. c and d, by two‐way ANOVA; f, h, j, k, and l, by unpaired t‐test. ^*^ *P* < 0.05; ^**^ *P* < 0.01; ^***^ *P* < 0.001; ns, no significance. KO: CTHRC1 knockout; AAV‐C1: adeno‐associated virus overexpressing CTHRC1; AAV‐Ctr: empty AAV vector; TI: tendon injury.

Adeno‐associated virus overexpressing CTHRC1 (AAV‐C1) was injected in situ into the Achilles tendons of CTHRC1 KO mice to construct an overexpression model. CTHRC1 was significantly overexpressed 3 weeks after injection and maintained at a high level (Figure S17, Supporting Information). Subsequently, to further determine whether overexpression of CTHRC1 could reverse the impaired tendon repair ability in KO mice, we subjected the KO mice to the Achilles tendon injury 3 weeks after AAV‐C1 or empty AAV vector (AAV‐Ctr) injection (**Figure** **4**a). The ultrasound results showed that KO/AAV‐C1 mice had thinner tendons and their echo intensities were higher than those of KO/AAV‐Ctr mice at week 3 and 6 post‐injury, suggesting that overexpression of CTHRC1 reversed the poorer tendon repair effect caused by CTHRC1 deletion (Figure 4b–d). In addition, the MMP activity in KO/AAV‐C1 mice was found to be less pronounced compared to KO/AAV‐Ctr mice, suggesting overexpressing CTHRC1 could lead to a potentially favorable outcome in post‐injury recovery (Figure 4e,f).

Moreover, compared to KO/AAV‐Ctr mice, inflammatory cell infiltration, ground substance accumulation, and collagen fiber disorganization were improved in the Achilles tendons of KO/AAV‐C1 mice, which exhibited lower total Bonar scores (Figure 4g,h). KO/AAV‐C1 mice also had lower collagen type III percent in Achilles tendons than KO/AAV‐Ctr mice (Figure 4g,i). Thus, overexpression of CTHRC1 can reverse the impaired tendon histopathology mediated by CTHRC1 deletion in vivo.

Then, the mechanical assessments revealed a declined ultimate strain, an increased ultimate stress, and an increased Young's modulus in the Achilles tendons of KO/AAV‐C1 mice, compared to KO/AAV‐Ctr mice (Figure 4j). AFM analysis further demonstrated a higher microscopic Young's modulus in the KO/AAV‐C1 mice than that in KO/AAV‐Ctr mice (Figure 4k). Combined, the above results suggested that overexpression of CTHRC1 ameliorated both macroscopic and microscopic tendon biomechanical properties. Furthermore, there was a significant upregulation of the tenogenic marker expression levels in the Achilles tendons after overexpressing CTHRC1, indicating a stronger tenogenic capacity (Figures 4l and S18, Supporting Information).

In summary, these findings indicated that CTHRC1 overexpression ameliorates the worsened tendon repair induced by CTHRC1 deletion.

### 2.5 CTHRC1 Interacted with EGFR to Activate the MAPK Signaling Pathway

**Figure 5:**
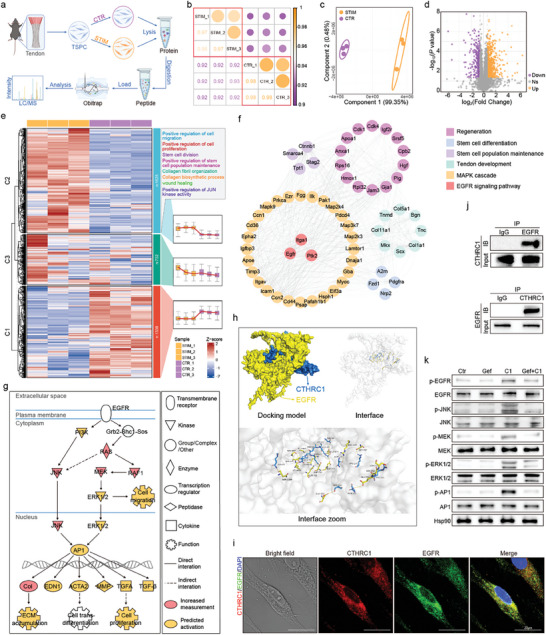
CTHRC1 functions by interacting with EGFR to activate the downstream MAPK signaling pathway. a) Schematic overview of proteomics on TSPC. b) Inter‐sample Spearman correlation heat map, with numbers representing correlation coefficient. c) PLS‐DA plot of the proteomic data. d) Volcano plot. e) Heatmap of protein expression profiles with GO biological process annotation of clusters. f) Protein‐protein interaction network. g) Ingenuity pathway analysis of DEPs. h) Illustrative diagram of molecular docking model. i) Colocalization of CTHRC1 and EGFR on TSPC by immunofluorescence. j) Cellular co‐immunoprecipitation assays of CTHRC1 and EGFR. k) Representative western blot images of indicated proteins in TSPC. TSPC: tendon stem/progenitor cell; MAPK: mitogen‐activated protein kinase; EGFR: epidermal growth factor receptor; IP: immunoprecipitation; IB: immunoblotting; C1: CTHRC1; Gef: gefitinib.

To explore the molecular mechanism of CTHRC1 on TSPC, we performed proteomic experiments by stimulating TSPC with rCTHRC1 (**Figure** **5**a). Both the correlation heatmap and PLS‐DA showed strong intra‐group correlations (Figure 5b,c), indicating the alteration of protein expression profiles. According to the volcano plot, many DEPs were observed between the control and CTHRC1‐stimulated groups (Figure 5d). Consistent with our above findings, the heatmap revealed enrichment of DEPs in cell migration and proliferation, stem cell division and population maintenance, collagen biosynthesis and organization, wound healing, and positive regulation of JUN kinase activity, revealing the effects of CTHRC1 on facilitating tendon healing (Figure 5e). Additionally, GO cell component analysis showed that DEPs were enriched in the cytoplasm, extracellular exosome, and nucleus, indicating active protein synthesis and secretion after CTHRC1 stimulation (Figure S19a, Supporting Information). Moreover, the GO molecular function terms of DEPs were associated with ATP binding, metal ion binding, and structural constituent of ribosome (Figure S19b, Supporting Information).

To find out the specific signaling pathway that mainly participated in the healing process, we further conducted an in‐depth bioinformatic analysis. PPI network analysis revealed that proteins referred to the EGFR pathway, MAPK pathway, regeneration, stem cell differentiation, stem cell population maintenance, and tendon development were closely related, which positively participated in tendon healing (Figure 5f). PPI using the proteomic data from the in vivo injury model also illustrated the important role of EGFR/MAPK pathway in tendon injury (Figure S3c, Supporting Information). Moreover, ingenuity pathway analysis (IPA) demonstrated that CTHRC1 activated the MAPK signaling pathway via binding to the EGFR in TPSC (Figure 5g).

To further elucidate that CTHRC1 could interact with EGFR to activate downstream MAPK signaling, we performed a protein‐protein docking model. The molecular docking demonstrated that CTHRC1 and EGFR could firmly form hydrogen bonds on the interaction interface, with a binding energy of −10.1 kcal mol^−1^ and interface area of 2873.1 Å^2^, indicating strong binding affinity between CTHRC1 and EGFR (Figures 5h and S20a and Table S1, Supporting Information). Additionally, colocalization of CTHRC1 and EGFR was found among TSPC by immunofluorescence (Figure 5i). What's more, co‐immunoprecipitation also confirmed the interaction between CTHRC1 and EGFR (Figure 5j). Finally, the GST pull‐down assay demonstrated that CTHRC1 directly interacted with EGFR (Figure S20b, Supporting Information). In summary, the above jointly illustrated that CTHRC1 could combine with EGFR.

Moreover, we applied gefitinib as an EGFR inhibitor to confirm that CTHRC1 mainly activated MAPK signaling through binding to EGFR.^[^ ^44^ ^]^ After adding rCTHRC1 to TSPC, we observed increased phosphorylation levels of EGFR and MAPK signaling pathway molecules. However, upon the addition of gefitinib co‐stimulation, the phosphorylation levels of EGFR and MAPK signaling pathway molecules were significantly downregulated, confirming that CTHRC1 could activate the downstream MAPK signaling pathway by binding to EGFR (Figures 5k and S21, Supporting Information). Similarly, CTHRC1 deletion significantly suppressed the phosphorylation of EGFR and MAPK signaling pathway molecules in the injured Achilles tendons (Figure S22, Supporting Information). Collectively, these results indicated that CTHRC1 could interact with EGFR to further activate the downstream MAPK signaling pathway during tendon healing response.

### 2.6 EGFR/MAPK Inhibitor Reverses the Positive Effects of CTHRC1 on TSPC In Vitro

**Figure 6:**
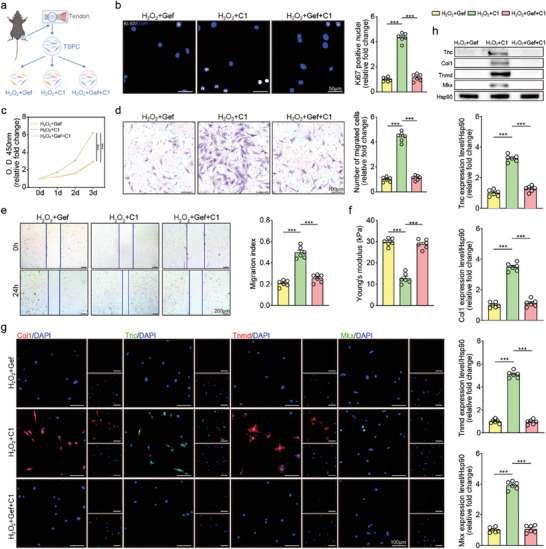
Gefitinib (Gef) reverses the effects of CTHRC1 on the proliferation, migration, and tenogenic differentiation of TSPC in vitro. a) Schematic of the experimental protocol. b) Representative immunofluorescent images of proliferative TSPC with Ki67‐labeled nuclei and quantification (n = 6). c) TSPC proliferation curves at indicated time (n = 6). d) Representative images of transwell assay and quantification (n = 6). e) Representative images and corresponding quantification of wound healing assay (n = 6). f) Quantification of Young's modulus by AFM analysis (n = 6). g) Representative immunofluorescent micrographs of indicated proteins in TSPC. h) Representative western blot images of indicated proteins in TSPC and quantification (n = 6). Data are presented as mean ± SEM. c, by two‐way ANOVA; b, d, e, f, and h, by one‐way ANOVA. ^***^ *P* < 0.001. TSPC: tendon stem/progenitor cell; C1: CTHRC1; Gef: gefitinib.

To further investigate whether CTHRC1 exerted its effects on TSPC through EGFR/MAPK signaling pathway, we used gefitinib in vitro as previously described^[^ ^44^ ^]^ (**Figure** **6**a). According to CCK‐8 and Ki67 immunofluorescence assays, the addition of gefitinib significantly inhibited the enhancement of TSPC proliferation stimulated by CTHRC1, indicating that inhibiting EGFR could counteract the proliferative effect of CTHRC1 on TSPC (Figure 6b,c). Next, we conducted the transwell and wound healing assays to investigate the effect on TSPC migration, finding that the enhanced migratory capacity of TSPC by CTHRC1 was abrogated by the EGFR inhibitor (Figure 6d,e). Besides, after treatment with gefitinib, a significant increase in the microscopic Young's modulus of TSPC was detected by AFM, implying that the promotion of cell motility by CTHRC1 could be effectively abolished by blocking EGFR (Figures 6f and S23, Supporting Information).

Moreover, CTHRC1‐induced promotion of tenogenic marker mRNA expression in TSPCs was reversed by the EGFR inhibitor (Figure S24, Supporting Information). Both the immunofluorescence and western blot assays showed that a reversal of the increased tenogenic protein expression level by CTHRC1 occurred after inhibiting EGFR (Figures 6g,h and S25, Supporting Information). In brief, these suggested that inhibition of EGFR could reverse the boosting tenogenic effect of CTHRC1 on TSPC.

Additionally, we further examined the impact of MAPK inhibition on TSPC. Similarly, MAPK inhibitor reversed the effects of CTHRC1 on the proliferation, migration, and tenogenic differentiation of TSPC in vitro (Figure S26a‐d, Supporting Information).

Overall, the above results illustrated that blocking EGFR/MAPK can reverse the effects of CTHRC1 on the proliferation, migration, and tenogenic differentiation of TSPC in vitro, indicating that CTHRC1 exerts its functions on TSPC through the EGFR/MAPK pathway.

### 2.7 EGFR Inhibitor Abrogates the Tendon‐Healing Property of CTHRC1

**Figure 7:**
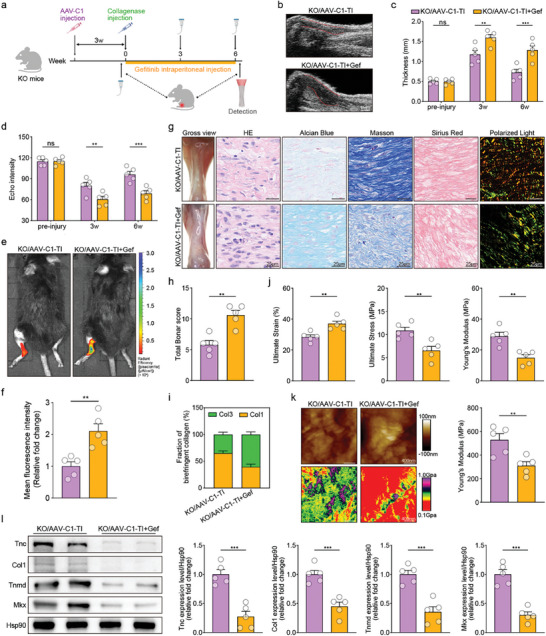
Gefitinib (Gef) reverses the tendon repair of KO/AAV‐C1 mice. a) Diagram of the experimental approach. b) Representative ultrasound images of Achilles tendon at week 6 post‐injury. c) Assessment of Achilles tendon thickness at different time points (n = 5). d) Assessment of echo intensity at different time points (n = 5). e) Representative in vivo fluorescence imaging. f) Quantification of mean fluorescence intensity (n = 5). g) Representative images of gross view, HE, Alcian Blue, Masson, Sirius Red staining, and Sirius Red staining using polarized light microscopy. h) Assessment of Achilles tendon using total Bonar score (n = 5). i) Fraction of birefringent collagen in Sirius Red staining imaged under polarized light (n = 5). j) Assessment of ultimate strain, ultimate stress, and Young's modulus of Achilles tendon (n = 5). k) Representative AFM images of Achilles tendon and assessment of Young's modulus by AFM analysis (n = 5). l) Representative western blot images of indicated proteins in Achilles tendon and quantification (n = 5). Data are presented as mean ± SEM. c and d, by two‐way ANOVA; f, h, j, k, and l, by unpaired t‐test. ^**^ *P* < 0.01; ^***^ *P* < 0.001; ns, no significance. KO: CTHRC1 knockout; AAV‐C1: adeno‐associated virus overexpressing CTHRC1; TI: tendon injury; Gef: gefitinib.

To further verify the role of EGFR in CTHRC1 facilitating tendon healing, we administrated gefitinib into the KO/AAV‐C1 mice post‐injury intraperitoneally^[^ ^44^ ^]^ (**Figure** **7**a). Ultrasound revealed that compared to KO/AAV‐C1 mice, thicker tendon and lower echo intensity was observed after treating with gefitinib at week 3 and 6 post‐injury, indicating that inhibiting EGFR reversed the enhanced tendon healing by overexpression of CTHRC1 in KO mice (Figure 7b–d). Additionally, KO/AAV‐C1+Gef mice had increased MMP activity detected by in vivo imaging, which was unfavorable for tendon healing (Figure 7e,f).

Besides, histological examination showed more severe inflammatory cell infiltration, ground substance accumulation, and disordered collagen fiber organization after inhibiting EGFR, exhibiting significantly higher total Bonar scores and a greater percentage of collagen type III percent (Figure 7g–i). These results illustrated the histopathologic improvement due to overexpressing CTHRC1 in KO mice was reversed by EGFR inhibitor.

Next, Achilles tendons in KO/AAV‐C1+Gef mice exhibited diminished mechanical strength, as evidenced by a rise in ultimate strain, a decrease in ultimate stress, and a decline in Young's modulus (Figure 7j). In parallel, lower microscopic Young's modulus, reflecting a weaker microscopic mechanical strength, was observed in the KO/AAV‐C1+Gef mice (Figure 7k). Consequently, inhibition of EGFR negated the improvement in macroscopic and microscopic tendon biomechanical properties brought about by CTHRC1. Additionally, the tenogenic marker expression levels in the Achilles tendons of KO/AAV‐C1+Gef mice were significantly lower than those in KO/AAV‐C1 mice (Figure S27, Supporting Information). Consistently, similar findings could be observed in the western blot (Figure 7l). Accordingly, it could be seen that the blockade of EGFR nullified the enhancement in the tenogenic capacity induced by CTHRC1. Briefly, these findings manifested that blocking EGFR could abolish the enhanced tendon repair effect mediated by CTHRC1, indicating that CTHRC1 promoted the repair response via the EGFR pathway in the injured tendon.

## 3 Discussion

**Figure 8:**
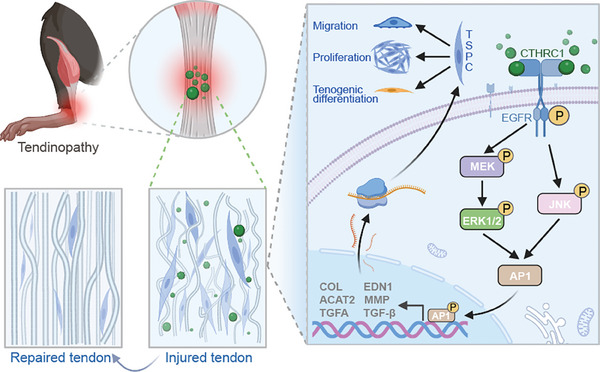
Schematic illustration of effects of CTHRC1 on tendon healing.

In this study, we identify that CTHRC1 may serve as a potential effector in promoting tendon healing after injury via multi‐proteomic analysis. CTHRC1 knockdown impairs the tendon healing process, while overexpression of CTHRC1 promotes tendon healing after injury. Specifically, CTHRC1 interacts with EGFR and subsequently activates the MAPK signaling pathway to promote TSPC proliferation, migration, and tenogenic differentiation, thus promoting tendon regeneration and attenuating tendinopathy (**Figure** **8**). Our findings suggest that CTHRC1 offers a novel promising therapeutic strategy for tendinopathy.

Tendon regeneration is critical to the successful treatment of tendinopathy, but it also poses a great challenge due to incompetent autonomous regeneration of the tendon. Developing strategies for tissue regeneration requires a better understanding of developmental mechanisms.^[^ ^4^, ^45^ ^]^ In echo of this, there is a remarkable tendon regeneration capacity in newborn mice.^[^ ^5^, ^12^ ^]^ To identify proteins involved in tendon regeneration, we conducted a proteomics experiment and detected a substantial upregulation of CTHRC1. Similarly, CTHRC1 was also highly expressed in mice with tendon injury. The role of CTHRC1 in tissue repair has previously been noted in studies.^[^ ^22^, ^24^, ^25^, ^26^, ^27^, ^28^ ^]^ Therefore, we initially speculated CTHRC1 as a key factor in tendon regeneration, which possesses potential properties in tendon healing.

Our study found that CTHRC1 exerted a beneficial influence on TSPC. Previous studies have demonstrated the crucial role of TSPC in tendon regeneration, and there is growing interest in TSPC as a promising approach for tendon injury.^[^ ^19^, ^46^, ^47^, ^48^, ^49^ ^]^ However, the challenge in applying TSPC for therapeutic purposes lies in how to ensure sufficient quantities to prevent the depletion of TSPC repertoire, and how to enhance its tenogenic differentiation. Considering the scarcity of endogenous TSPC and the pauci‐cellular distribution of tendons, the insufficient activation of resident TSPC upon injury may hinder the healing process.^[^ ^20^, ^50^ ^]^ The stimulatory effects of CTHRC1 on TSPC proliferation and migration are crucial for enhancing the rapid increase and maintenance of the TSPC population at the injury site, which is of significant necessity for tendon regeneration. Another outstanding challenge that remains is the precise orchestration of TSPC tenogenic differentiation.^[^ ^51^ ^]^ The compelling need for enhancing TSPC tenogenic differentiation in tendon regeneration is evident, as it facilitates the formation of functional new tissue to regenerate damaged tendons, thereby accelerating the healing process and restoring tissue homeostasis. Our study showed that CTHRC1 could enhance TSPC tenogenic potential. Moreover, passaging has a significant impact on TSPC characteristics, particularly the weakened tenogenic ability and stemness.^[^ ^19^, ^52^ ^]^ We discovered that CTHRC1 maintained the number and stemness of TSPC during cell passaging, which holds significant implications for regenerative medicine and tissue engineering.^[^ ^36^, ^37^ ^]^ Overall, the beneficial effect of CTHRC1 on TSPC effectively helps overcome the limitations of current TSPC therapy, and it is significant for enhancing tendon regeneration.

Given that TSPC is essential for tendon regeneration after injury, we propose that CTHRC1 might play an active part in tendon healing. Through in vivo experiments, we confirmed that CTHRC1 knockdown impaired tendon healing in mice. The deleterious effects are multifaceted, encompassing tendon morphology, MMP activity, histopathology, biomechanics at the macro and micro levels, and tenogenic capacity. Conversely, overexpressing CTHRC1 can ameliorate the detrimental impact on tendon healing. These results strongly prove that CTHRC1 can enhance tendon healing and attenuate tendinopathy.

To further investigate the molecular mechanism by which CTHRC1 promotes TSPC proliferation, migration, and tenogenic differentiation, we conducted proteomics on CTHRC1‐stimulated TSPC. As a result, the MAPK pathway was identified as the primary signaling pathway and was highly activated. MAPK has been reported to play a crucial role in tendon development and repair.^[^ ^53^, ^54^, ^55^, ^56^ ^]^ It is also associated with TSPC proliferation and migration^[^ ^57^ ^]^ and anti‐inflammatory responses.^[^ ^58^ ^]^ Furthermore, our study identified EGFR as a receptor of CTHRC1 in TSPC. EGFR, a member of the receptor tyrosine kinase and the HER/ErbB protein family, has MAPK as one of its downstream pathways.^[^ ^59^ ^]^ The EGFR/MAPK pathway is essential for tissue repair and healing.^[^ ^60^, ^61^ ^]^ Our studies confirmed that blocking EGFR significantly suppressed the favorable impact of CTHRC1 on TSPC, regarding proliferative, migrative, and tenogenic capability. Similarly, inhibiting EGFR markedly counteracted the augmented tendon healing effect induced by CTHRC1. These results demonstrated that CTHRC1 activates TSPC primarily by binding to EGFR and activating the downstream MAPK pathway, thereby promoting tendon healing.

Our study demonstrated the crucial role of CTHRC1 in tendon regeneration. The tendon healing process typically consists of three phases: inflammation (spanning days), proliferation (spanning weeks), and remodeling (lasting for months).^[^ ^32^, ^42^ ^]^ The slow transition process lasts over a year. However, our findings revealed that although CTHRC1 level transiently increased after tendon injury in the natural course, it consistently declined within a few weeks. This is inadequate for a protracted repair process and limits the capacity of tendon regeneration. Consequently, we believe that supplementation of CTHRC1, with the aim to maintain its consistently high levels during tendon healing, holds promise as a potential therapeutic approach for tendinopathy.

The present study has some limitations. First, the extent to which rodent models of collagenase‐induced tendinopathy accurately represent human conditions remains uncertain. Second, this study doesn't employ conditional CTHRC1 KO mice to more convincingly investigate the specific effect of CTHRC1 in tendinopathy. Third, the precise molecular mechanism of CTHRC1 may differ between mice and humans, so further preclinical trials are needed before CTHRC1 can be applied to humans. Nevertheless, our study has preliminarily unveiled the crucial role of CTHRC1 in tendon regeneration, holding promise for clinical translation.

In summary, our work reveals a novel role of CTHRC1 in promoting tendon healing, which it achieves by enhancing the proliferation, migration, and tenogenic capacity of TSPC. Mechanistically, CTHRC1 interacts with EGFR, leading to the activation of the MAPK signaling pathway and subsequently facilitating the tendon healing process. Our study holds great translational value for the treatment of tendinopathy.

## Conflict of Interest

The authors declare no conflict of interest.

## Author Contributions

C.C., X.Z., and C.W. contributed equally to this work. C.C. and T.Y. conceptualized the study. C.C. and C.W. drafted the manuscript. C.C., T.Y., X.Z., and C.W. performed the study. C.C., X.Z., and Y.Z. reviewed the literature. C.C., H.Z., and T.Y. analyzed the data. Y.Y. provided project administration and supervision. Y.Z., T.Y., and Y.Y. revised the manuscript. All authors read and approved the final version.

## Supporting information



Supporting Information

## Data Availability

The data that support the findings of this study are available from the corresponding author upon reasonable request.
