# Supplementary material for: CTHRC1 Attenuates Tendinopathy via Enhancing EGFR/MAPK Signaling Pathway
Source: Adv Sci (Weinh). 2024 Nov 14;11(47):2406611. doi: 10.1002/advs.202406611 (PMC11653680; doi:10.1002/advs.202406611)
Supplement: Supplementary file 1 — Supporting Information [file ADVS-11-2406611-s001.docx]

Supporting Information

**CTHRC1 Attenuates Tendinopathy via Enhancing EGFR/MAPK Signaling Pathway**

*Cheng Chen, Xu Zheng, Cheng Wang, HaiChao Zhou, Yi Zhang, TianBao Ye, YunFeng Yang*

**This PDF file includes:**

Experimental Section

Figure S1 to S28

Table S1

**Experimental Section**

**Animal**

This study used wild-type (WT) C57BL/6 male mice aged 6-8 weeks purchased from Shanghai SLRC Experimental Animal Co., Ltd. (Shanghai, China). CTHRC1 knockout (KO) mice on a C57BL/6J genetic background were generated by Riken BioResource Center. Briefly, the coding sequence of the first exon in Collagen triple helix repeat-containing 1 (CTHRC1) was replaced with LacZ via a targeting vector constructed by a cassette consisting of LacZ-pA and PGK-neopA^[1-2]^. The mice were raised in a specific pathogen-free environment. Animal experiments were strictly conducted in accordance with the regulations of the Animal Care and Use Committee of Shanghai Jiao Tong University. In addition, all animal procedures followed the guidelines for the care and use of laboratory animals set forth by the National Institutes of Health and approved by the Animal Care and Use Committee of Shanghai Jiao Tong University. Euthanasia of mice was carried out by inhalation of carbon dioxide followed by cervical dislocation.

**Experimental tendinopathy mouse model**

The mouse tendinopathy model was established by injecting collagenase type I based on the previously described^[3]^. Briefly, the male C57BL/6J mice were anesthetized and injected the collagenase type I (125U, Sigma) into the Achilles tendon under ultrasound guidance using a 32-gauge insulin syringe.

**Adeno-associated virus (AAV) for mice**

AAV overexpressing CTHRC1 (AAV-C1) and empty AAV vector (AAV-Ctr) were purchased from Hanbio Biotechnology (Shanghai, China). AAV-CTHRC1 and AAV-Ctr (2 × 10^9^ particles in 2 μL) were injected into the Achilles tendon under ultrasound guidance three weeks before modeling, and validation was performed to ensure the effectiveness of AAV.

**Gefitinib administration** **for mice**

Mice were administered with gefitinib (Sigma) daily via intraperitoneal injection at a dosage of 100 mg/kg^[4]^. Animals receiving saline alone served as the control group. The treatment commenced at the first day of the injection of collagenase type I (125U, Sigma), then daily thereafter and continued for six weeks.

**Primary culture of mouse** **tendon stem/progenitor cell (TSPC)**

TSPCs were extracted as previously described^[5]^. In brief, the excised Achilles tendon was finely chopped and enzymatically digested using 3 mg/ml collagenase type I (ThermoFisher) and 4 mg/ml dispase (Roche) at 37°C for 40 minutes. Subsequently, the digested tissue was filtered through a 70-μm strainer to obtain single-cell suspensions, which were then cultured in Dulbecco's modified Eagle's medium (DMEM) with 20% fetal bovine serum (FBS, ThermoFisher), 2mM L-glutamine (ThermoFisher), and 100 U/ml penicillin/streptomycin (ThermoFisher). When TSPCs formed colonies, they were verified by flow cytometry analysis. Once confirmed as TSPCs, they were collected, expanded, and used for subsequent cell experiments. The cells were incubated at 37 ℃ in 5% CO_2_.

**Cellular intervention**

To emulate the injury environment, TSPCs were subjected to a 4-hour incubation with 200 μM hydrogen peroxide (H_2_O_2_, Sigma), within a medium composed of DMEM devoid of serum components^[5]^. TSPCs were incubated with recombinant human CTHRC1 protein (rCTHRC1, 1000ng/ml, MCE) upon stimulation. CTHRC1 neutralizing antibody (Sigma) was used to abrogate the function of CTHRC1. Gefitinib (20 nM, Sigma) was used to block the EGFR on TSPCs^[6]^. MAPK-IN-1 (Compound 2) (10 μM, MCE) was used to inhibit the MAPK signaling pathway in TSPCs^[7]^.

**Flow cytometry**

TSPCs were blocked with Fc Block (BioLegend), then stained with CD90 (BioLegend), CD44 (BioLegend), CD45 (BioLegend), and CD34 (BioLegend) for 30 minutes in the dark. The samples were washed twice and then analyzed using a flow cytometer (BD FACSA). The data were analyzed using Flowjo (v10.8.1)

**Proteomics**

The label-free proteomics analysis was conducted following the previous study^[8]^. Samples were lysed with RIPA buffer (Beyotime) containing proteinase and phosphatase inhibitors (Beyotime) on ice. Subsequently, proteins were precipitated with acetone at -20°C overnight, followed by reduction with DTT (ThermoFisher) at 55°C for 1 hour and alkylation with IAA (ThermoFisher) at room temperature in the dark for 30 minutes. Protein digestion was carried out overnight at 37°C using Trypsin Protease (ThermoFisher), and the reaction was terminated with FA (ThermoFisher). The resulting peptides were desalted using a MacroSpin C18 column (Nest Group), lyophilized in a vacuum concentrator, reconstituted in 0.1% FA, and quantified using a ThermoFisher quantitative fluorometric peptide assay prior to LC-MS/MS analysis. For data-independent analysis (DIA), peptides from each sample were fractionated using a high-pH reversed-phase spin column (ThermoFisher) as per the manufacturer's instructions. Peptide identification was performed using a Q-Exactive^TM^ Plus Hybrid Quadrupole-Orbitrap^TM^ mass spectrometer (ThermoFisher), and the MS raw files were analyzed with Maxquant. Differentially expressed proteins were subjected to Gene Ontology (GO) term analysis using DAVID (https://david.ncifcrf.gov/). Gene Set Enrichment Analysis (GSEA) was carried out using GSEA (v4.3.2). Protein and protein interaction (PPI) analysis was performed via the String database (https://cn.string-db.org/) and visualized using Cytoscape (v 3.8.0). Ingenuity pathway analysis (IPA) upstream regulator analysis was conducted via IPA (Qiagen).

**Quantitative real-time polymerase chain reaction (****qRT-PCR)**

Total RNA was extracted from tissues or cells utilizing the Total RNA Isolation Kit (Vazyme) following the manufacturer's instructions. Subsequently, cDNA synthesis was carried out using cDNA Synthesis Kit (Vazyme) according to the manufacturer's protocol. qRT-PCR was performed using the Universal SYBR Green Master Mix (Vazyme). 18s served as the housekeeping gene. The primer sequences for qRT-PCR were as follow: *18s*: 5'- CCTGTATGTGGCTGTGACTC -3', 5'- TCAAAGTAAGACCTCCGAATGG -3'; *Cthrc1*: 5'- CAGTTGTCCGCACCGATCA -3', 5'- GGTCCTTGTAGACACATTCCATT -3'; *Oct4*: 5'- CGGAAGAGAAAGCGAACTAGC -3', 5'- ATTGGCGATGTGAGTGATCTG -3'; *Sox2*: 5'- GCGGAGTGGAAACTTTTGTCC -3', 5'- CGGGAAGCGTGTACTTATCCTT -3'; *Col1a1*: 5'- GCTCCTCTTAGGGGCCACT -3', 5'- CCACGTCTCACCATTGGGG -3'; *Tnmd*: 5'- ACACTTCTGGCCCGAGGTAT -3', 5'- GACTTCCAATGTTTCATCAGTGC -3'; *Scx*: 5'- CTGGCCTCCAGCTACATTTCT -3', 5'- GTCACGGTCTTTGCTCAACTT -3'; *Tnc*: 5'- ACGGCTACCACAGAAGCTG -3', 5'- ATGGCTGTTGTTGCTATGGCA -3'; *Bgn*: 5'- TGCCATGTGTCCTTTCGGTT -3', 5'- CAGGTCTAGCAGTGTGGTGTC -3'; *Mkx*: 5'- GGGGAGCCGTGCTTTTTGA -3', 5'- GCCTTACCTTCCCTCCATTCTG -3'. Gene expression levels were calculated using the standard comparative CT method.

**Immunofluorescence staining**

For the immunofluorescence staining of tendon tissue, freshly harvested tendons were embedded in optimum cutting temperature (OCT) compound and then cut into 10 μm sections. After fixation and permeabilization, the cryosections were blocked with 10% goat serum albumin and 0.03% TritonX-100 for 1 hour at room temperature. Subsequently, cryosections were incubated overnight at 4 ℃ with indicated primary antibodies. After washing with PBS, the cryosections were stained with corresponding fluorescence secondary antibodies for 2 hours at room temperature. Then, nuclear was stained with 4',6-diamidino-2-phenylindole (DAPI) (Invitrogen). Antifluorescence quencher was used to seal the sections.

For the immunofluorescence co-localization assay of TSPC, TSPC was treated with 1000 ng/ml rCTHRC1 (MCE) for 10 minutes. For the immunofluorescence staining of cells, the cells were subjected to fixation using a 4% solution of paraformaldehyde. Subsequently, the cells were permeabilized with a 0.03% Triton X-100 solution for 20 minutes. The cells were then blocked with a 5% bovine serum albumin (BSA) solution containing 1% donkey serum for 30 minutes. The cells were further incubated with specific primary antibodies at 4°C overnight. This was followed by a 1-hour incubation at room temperature with corresponding fluorescence secondary antibodies. Nuclear was stained by DAPI (Invitrogen). Antifluorescence quencher was used to seal the sections. The primary antibodies used were as follow: CTHRC1 (1:50, Proteintech); CD90 (1:250, Abcam); CD45 (1:100, Abcam); CD31 (1:50, Abcam); Tnc (1:50, ThermoFisher); Col1a1 (1:200, Abcam); Tnmd (1:100, ThermoFisher); Mkx (1:100, Santa Cruz); EGFR (1:250, Abcam). The fluorescence was captured using a fluorescence microscope. The intensity of the fluorescence was quantitatively analyzed employing ImageJ (v 1.53t).

**Western blot**

Total proteins were extracted from tissues or cells by employing RIPA buffer supplemented with complete protease inhibitor (Roche) and phosphatase inhibitor (Roche, Germany). The concentration of proteins was determined utilizing the Pierce BCA Protein Assay Kit (ThermoFisher). Equal amounts of protein were then loaded into SDS/PAGE gels. Following electrophoresis, proteins from the SDS/PAGE gels were transferred to Polyvinylidene difluoride (PVDF) membranes and blocked with 5% BSA/TBS. The membranes were then incubated overnight with specific primary antibodies at 4°C. The primary antibodies used were as follow: HSP90 (1:10000, Proteintech; 1:20000, Abcam); CTHRC1 (1:500, Proteintech); Oct4 (1:1000, Proteintech); Sox2 (1:500, Proteintech); Tnc (1:200, ThermoFisher); Col1a1 (1:1000, Abcam); Tnmd (1:500, ThermoFisher); Mkx (1:200, Santa Cruz); p-EGFR (1:1000, Abcam); EGFR (1:1000, Abcam); p-JNK (1:1000, Abcam); JNK (1:1000, Abcam); p-MEK (1:1000, Abcam); MEK (1:1000, Abcam); p-ERK1/2 (1:1000, Cell Signaling Technology); ERK1/2 (1:1000, Cell Signaling Technology); p-AP1 (1:1000, Abcam); AP1 (1:500, Abcam). Subsequently, the membranes were incubated with a horseradish peroxidase-conjugated secondary antibody for 2 hours at room temperature. Protein bands on the membranes were visualized using a Tanon instrument and quantified using ImageJ (v 1.53t).

**Enzyme-linked immunosorbent assay (ELISA) assay**

The concentration of CTHRC1 was assessed using an ELISA kit from CUSABIO. Tendon tissue samples were prepared by treating them with ice-cold Lysis Buffer (10 mM Tris (pH 8.0), 130 mM NaCl, 1% TritonX-100, and protease inhibitor cocktail) for 60 minutes, followed by centrifugation to collect the supernatant. The measurements were performed according to the manufacturer's instructions, and the absorbance at 450 nm was monitored using a microplate reader (BioTek).

**Ultrasound imaging**

Ultrasound imaging was utilized to assess tendon thickness and echo intensity in this study. The ultrasound system employed was Vevo 2100 instrument (Visualsonic) equipped with an MS-400 probe. The images in B-mode were acquired. The thickness and echo intensity of ROI were measured by Vevo Lab (v 5.7.1).

**Histology**

Upon collection, the tendons were promptly immersed in a solution of 4% paraformaldehyde to facilitate fixation. Subsequently, they underwent a process of dehydration through a gradient alcohol, culminating in embedding within paraffin blocks. Thereafter, microtomy was employed to produce histological sections of 5 μm in thickness. Hematoxylin and Eosin (HE), Alcian Blue, Masson's trichrome, and Sirius Red staining were performed according to the manufacturer’s instructions (Sigma). The histological grading of the tissue samples was conducted employing a refined version of the Bonar scoring system, which ranges from 0 to 12^[3, 9]^. Picrosirius red staining was employed to visualize the collagen fiber architecture under polarized light microscopy. Subsequently, the quantitative analysis of the collagen subtypes was conducted utilizing the ImageJ (v 1.53t).

**Atomic force microscopy (AFM)**

The microscopic mechanical properties of cell and tissue were examined using AFM (FastScan Bio, Bruker, USA). To analyze the surface morphology of tissues, an AC240 probe was employed with a scanning rate of 2.0 Hz, a peak force setpoint of 30 nN, and a peak force amplitude of 70 nm. The deflection sensitivity was calibrated based on the sapphire model, and data analysis was carried out using NanoScope Analysis (v 1.8). The Hertzian (Spherical) model was utilized to derive the apparent Young's modulus. Each scan produced a morphological image. To assess the microscopic mechanical properties of cells, an MLCT-O10 probe was utilized with a scanning rate of 1.0 Hz and a peak force amplitude of 100 nm. The force-distance curves obtained were analyzed using NanoScope Analysis (v 1.8). The force-distance curves were adjusted before applying the Hertzian (Spherical) model to extract the apparent Young's modulus. The sample Poisson's ratio was set to 0.3.

**Tendon biomechanical testing**

Biomechanical testing procedures were conducted on the Achilles tendon. Briefly, after the removal of surrounding soft tissues, the length and cross-sectional area of the tendons were measured using a high-precision ruler. Tendon mechanical properties were assessed using a tissue mechanics testing machine (Mach-1, Biomomentum, Canada). The Achilles tendon was securely fixed, and a pre-load of 0.01 N was applied. Each tendon underwent cyclic elongation with a load ranging from 0.02 to 0.04 N at a speed of 0.02 mm/s for 10 cycles. Subsequently, a failure test was conducted on the tendon at an elongation rate of 0.02 mm/s, during which the load-elongation behavior and failure mode were documented. The obtained load-displacement curves were analyzed using Mach-1 Analysis to determine the structural characteristics of the tendons.

**In vivo imaging**

Mice were initially anesthetized and shaved to minimize light scatter. The used probe was MMPSense 680 FAST (PerkinElmer), following the manufacturer's guidelines. The probe was injected into the Achilles tendon. Subsequently, the mice were positioned within an IVIS SpectrumCT In Vivo Imaging System (PerkinElmer). The acquired imaging data were analyzed using Living Image (PerkinElmer).

**Cell Counting Kit-8 (CCK-8) assay**

TSPCs (1 × 10^4^ cells/well) were seeded in 96-well plates. Each group of cells was treated accordingly. TSPCs were subjected to a 4-hour incubation with 200 μM hydrogen peroxide (H_2_O_2_, Sigma). TSPCs were incubated with recombinant human CTHRC1 protein (rCTHRC1, 1000ng/ml, MCE) upon stimulation. CTHRC1 neutralizing antibody (Sigma) was used to abrogate the function of CTHRC1. Gefitinib (20 nM, Sigma) was used to block the EGFR on TSPCs^[6]^. MAPK-IN-1 (Compound 2) (10 μM, MCE) was used to inhibit the MAPK signaling pathway in TSPCs^[7]^. Cell viability was assessed using the CCK-8 (DOJINDO) according to the kit instructions. The absorbance was measured at 450 nm using a microplate reader (BioTek).

**Lactate dehydrogenase (LDH) assay**

TSPCs (1 × 10^4^ cells/well) were seeded in 96-well plates. Each group of cells was treated accordingly. TSPCs were subjected to a 4-hour incubation with 200 μM hydrogen peroxide (H_2_O_2_, Sigma). TSPCs were incubated with recombinant human CTHRC1 protein (rCTHRC1, 1000ng/ml, MCE) upon stimulation. CTHRC1 neutralizing antibody (Sigma) was used to abrogate the function of CTHRC1. Gefitinib (20 nM, Sigma) was used to block the EGFR on TSPCs^[6]^. MAPK-IN-1 (Compound 2) (10 μM, MCE) was used to inhibit the MAPK signaling pathway in TSPCs^[7]^. Cell cytotoxicity was assessed using the LDH Cytotoxicity Assay Kit (DOJINDO) according to the kit instructions. The absorbance was measured at 490 nm using a microplate reader (BioTek).

**Transwell assay**

Migratory assays were performed using an 8-μm transwell system (Corning). TSPCs were seeded at a density of 2 × 10^4^ cells per well in the upper chamber containing serum-free medium. Each group of cells was treated accordingly. TSPCs were subjected to a 4-hour incubation with 200 μM hydrogen peroxide (H_2_O_2_, Sigma). TSPCs were incubated with recombinant human CTHRC1 protein (rCTHRC1, 1000ng/ml, MCE) upon stimulation. CTHRC1 neutralizing antibody (Sigma) was used to abrogate the function of CTHRC1. Gefitinib (20 nM, Sigma) was used to block the EGFR on TSPCs^[6]^. MAPK-IN-1 (Compound 2) (10 μM, MCE) was used to inhibit the MAPK signaling pathway in TSPCs^[7]^. After 24 hours of incubation, non-migratory cells remaining in the upper chamber were removed. The migratory TSPCs were fixed with 4% paraformaldehyde, stained with 0.01% Crystal Violet (Sigma), and observed by microscopy.

**Wound** **healing** **assay**

TSPCs were seeded into 6-well plates to achieve a 90-100% confluence. Subsequently, a cellular wound was generated using a 200 μL pipette tip, and the culture medium was changed to fresh medium without FBS. To monitor the wound healing process, photographic documentation was performed microscopically at pre-determined time intervals of 0 hour and 24 hour post-wound induction. The quantification of the wound healing assay was conducted utilizing the ImageJ (v 1.53t).

**Coimmunoprecipitation**

TSPCs were incubated with rCTHRC1 for 10 minutes. Subsequently, the cells underwent a gentle wash with PBS followed by lysis using an immunoprecipitation lysis buffer (2.5 mM Tris (pH 7.4), 150 mM NaCl, 1 mM EDTA, 1% NP40, 5% glycerol, and a protease inhibitor cocktail). The co-immunoprecipitation procedure was carried out utilizing the Pierce Co-IP kit (ThermoFisher), following the manufacturer's instructions. Briefly, a total amount of 10 μg antibodies targeting CTHRC1 or epidermal growth factor receptor (EGFR) were transferred to AminLink Plus Coupling Resin. The protein extracts were then loaded into the column and incubated overnight at 4°C. The eluted samples were subsequently subjected to analysis via western blotting. A negative control using IgG was included.

**GST pull-down assay**

GST pull-down assay was performed as previously described^[10]^. The purified GST‐tagged EGFR protein and control GST were bound with GST resin in PBS binding buffer for 3 h at 4°C. Following three washes with PBST buffer, an equivalent amount of His‐tagged CTHRC1 protein was introduced and rebound at 4°C overnight. Next, the rebound GST resins underwent five washes with PBST buffer and subsequently boiled in SDS loading buffer to dissociate the bound proteins for SDS‐PAGE. Western blotting was employed to identify the His-tagged CTHRC1 protein band.

**Molecular Docking Analysis**

The protein structural domains of CTHRC1 and EGFR were analyzed using the Protein Data Bank (PDB) database (http://www.rcsb.org/). The molecular docking of CTHRC1 and EGFR was performed using GRAMM-X (http://gramm.compbio.ku.edu/). Pymol (v 2.4) and PDBePISA (https://www.ebi.ac.uk/pdbe/pisa/) were used to delve deeper into the protein-protein interactions and to enhance the visual representation of these interactions.

**Statistical analysis**

All quantitative data were presented as mean ± standard error of mean (SEM). The samples or mice per group in each study are marked in the related figure legend (n). The normality of the data was assessed using the Shapiro-Wilk normality test. Unpaired t test or analysis of variance (ANOVA) test was used for normally distributed data. For non-normally distributed data, the Mann-Whitney test was employed. A *P*-value < 0.05 was considered statistically significant. Statistical analysis was performed using GraphPad Prism (v 9.0) and R (v 4.3.0).

**References**

[1] D. Wang, Y. Zhang, T. Ye, R. Zhang, L. Zhang, D. Shi, T. Li, G. Xia, K. Niu, Z. Zhao, Y. Chen, W. Pan, L. Liu, X. Jin, C. Shen, *Int J Biol Sci* **2023**, *19* (4), 1299.

[2] J. Li, Y. Wang, M. Ma, S. Jiang, X. Zhang, Y. Zhang, X. Yang, C. Xu, G. Tian, Q. Li, Y. Wang, L. Zhu, H. Nie, M. Feng, Q. Xia, J. Gu, Q. Xu, Z. Zhang, *EBioMedicine* **2019**, *40*, 43.

[3] Y. Cho, H. S. Kim, D. Kang, H. Kim, N. Lee, J. Yun, Y. J. Kim, K. M. Lee, J. H. Kim, H. R. Kim, Y. I. Hwang, C. H. Jo, J. H. Kim, *Sci Adv* **2021**, *7* (47), eabg6069.

[4] L. Xu, X. Li, F. Zhang, L. Wu, Z. Dong, D. Zhang, *Theranostics* **2019**, *9* (9), 2712.

[5] Y. Wang, S. Jin, D. Luo, D. He, C. Shi, L. Zhu, B. Guan, Z. Li, T. Zhang, Y. Zhou, C. Y. Wang, Y. Liu, *Nat Commun* **2021**, *12* (1), 1293.

[6] L. Wang, N. Liu, C. Xiong, L. Xu, Y. Shi, A. Qiu, X. Zang, H. Mao, S. Zhuang, *J Am Soc Nephrol* **2016**, *27* (9), 2631.

[7] J. S. Wu, Q. Y. Meng, X. H. Shi, L. X. Liu, Z. K. Zhang, H. S. Guan, C. L. Shao, C. Y. Wang, *Bioorg Chem* **2020**, *104*, 104246.

[8] L. Wang, A. Abdulla, A. Wang, A. R. Warden, K. Z. Ahmad, Y. Xin, X. Ding, *Anal Chem* **2022**, *94* (15), 6026.

[9] A. Fearon, J. E. Dahlstrom, J. Twin, J. Cook, A. Scott, *J Sci Med Sport* **2014**, *17* (4), 346.

[10] Y. W. Cheng, F. M. Zeng, D. J. Li, S. H. Wang, J. Z. He, Z. C. Guo, P. J. Nie, Z. Y. Wu, W. Q. Shi, B. Wen, X. E. Xu, L. D. Liao, Z. M. Li, J. Y. Wu, J. Zhan, H. Q. Zhang, Z. J. Chang, K. Zhang, L. Y. Xu, E. M. Li, *Cancer Commun (Lond)* **2021**, *41* (12), 1398.


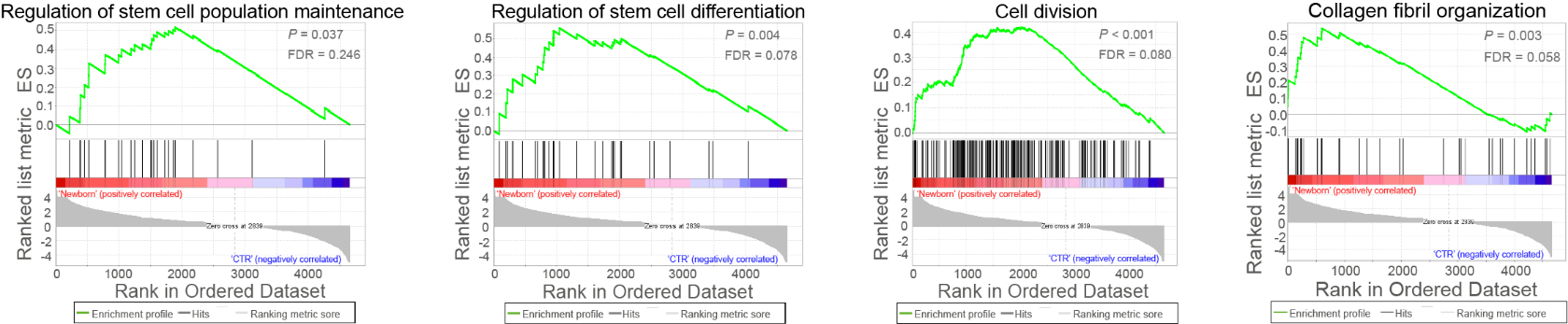


**Figure S1.** GSEA analysis (Newborn vs CTR) via GO biological process database. Adjusted *P* value and FDR are added to each enrichment dataset.


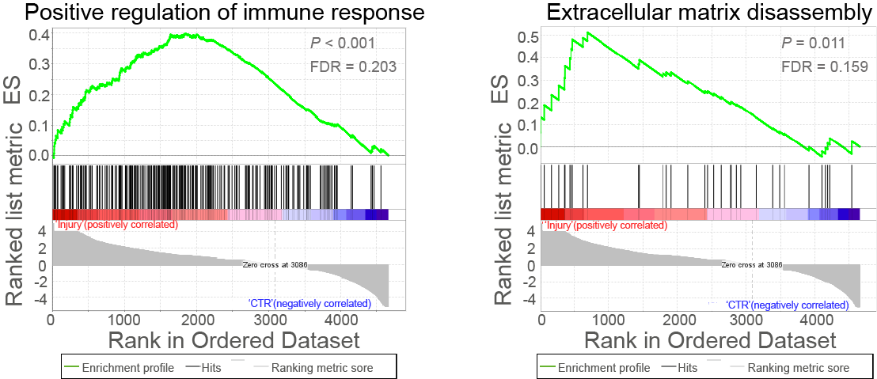


**Figure S2.** GSEA analysis (Injury vs CTR) via GO biological process database. Adjusted *P* value and FDR are added to each enrichment dataset.


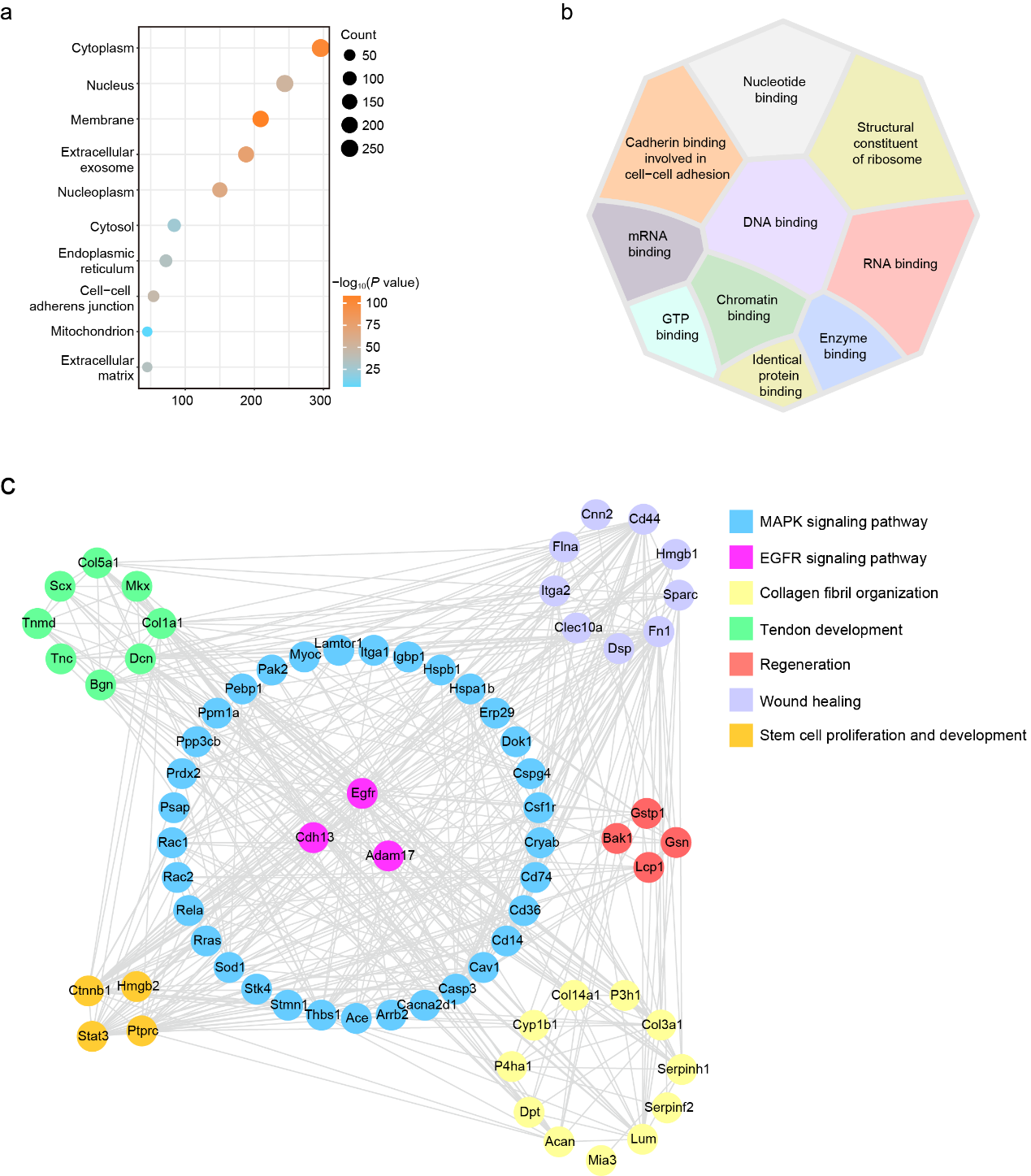


**Figure S3.** **a.** Bubble diagram of GO cell component. **b.** Enriched GO terms by molecular function. The area represents enrichment. **c.** Protein-protein interaction network using the proteomic data from in vivo injury model.


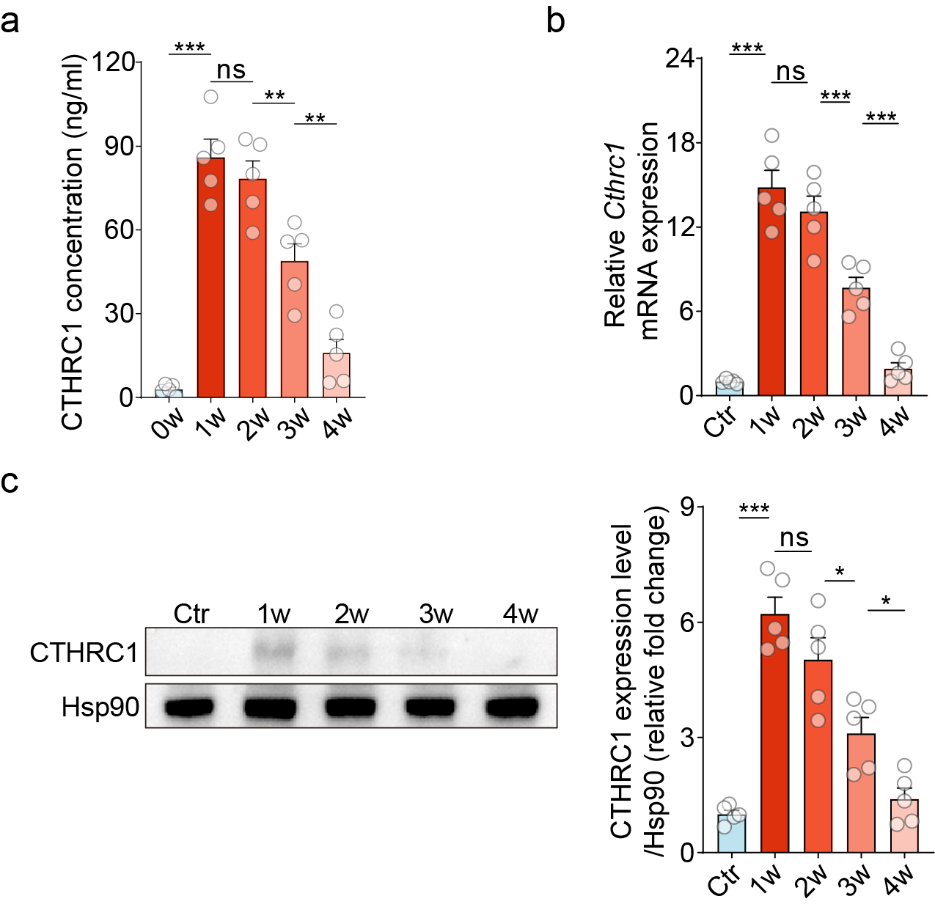


**Figure S4.** **The expression pattern of CTHRC1 in Achilles tendon after injury. a.** CTHRC1 levels in mice measured by ELISA (n = 5). **b.** Relative fold change of CTHRC1 mRNA expression in mice’s Achilles tendon of control and post-injury (n = 5). **c.** Representative western blot of CTHRC1 in mice’s Achilles tendon of control and post-injury, as well as quantifications (n = 5). Data are presented as mean ± SEM. One-way ANOVA was used. ^*^*P* < 0.05. ^**^*P* < 0.01. ^***^*P* < 0.001. ns, no significance.


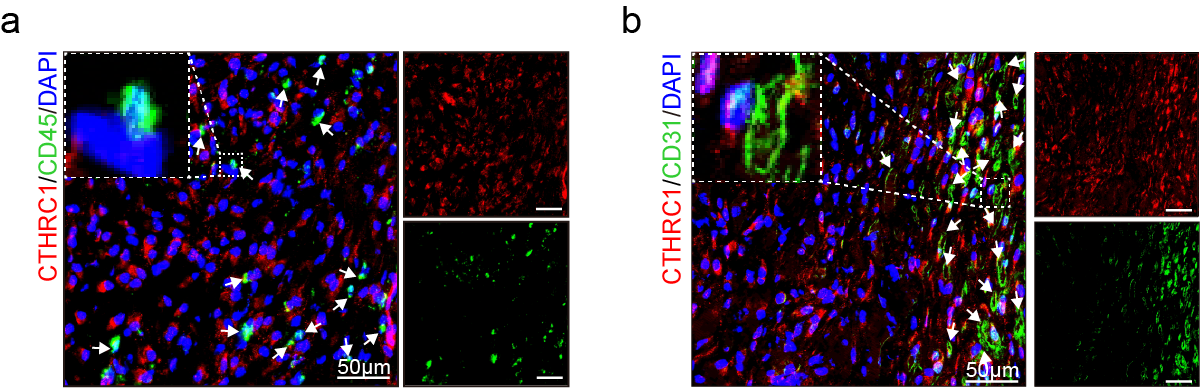


**Figure S5. a.** Colocalization of CTHRC1 and CD45 in tendon tissue by immunofluorescence. **b.** Colocalization of CTHRC1 and CD31 in tendon tissue by immunofluorescence.


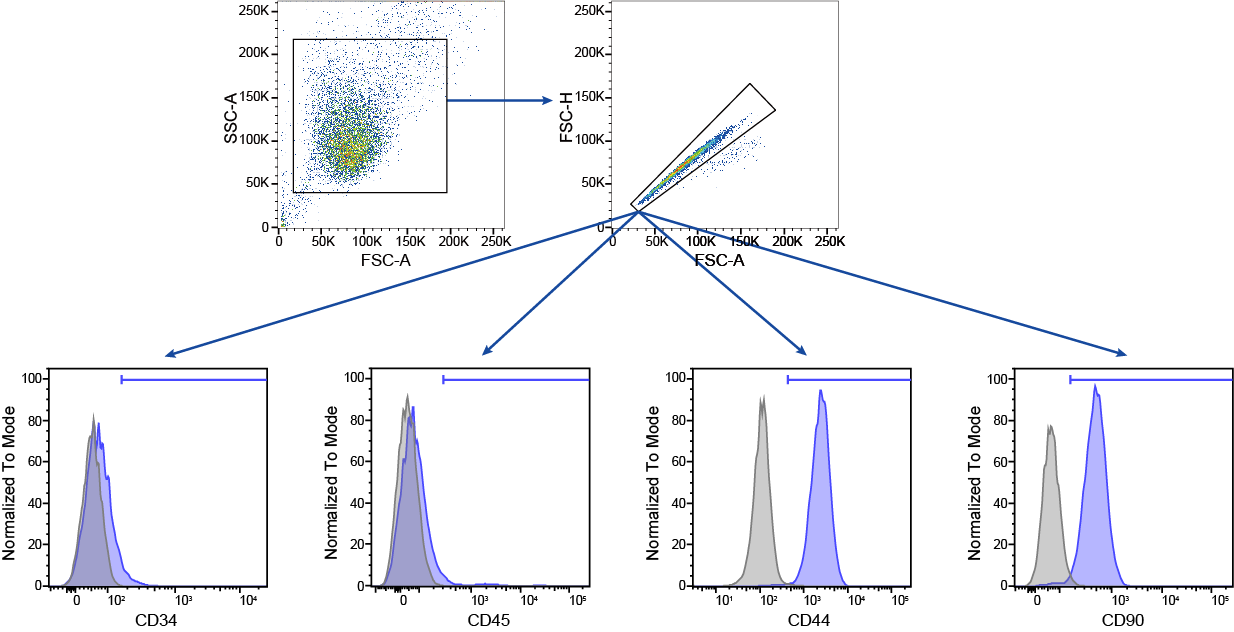


**Figure S6** Flow cytometric gating strategy to identify TSPC.


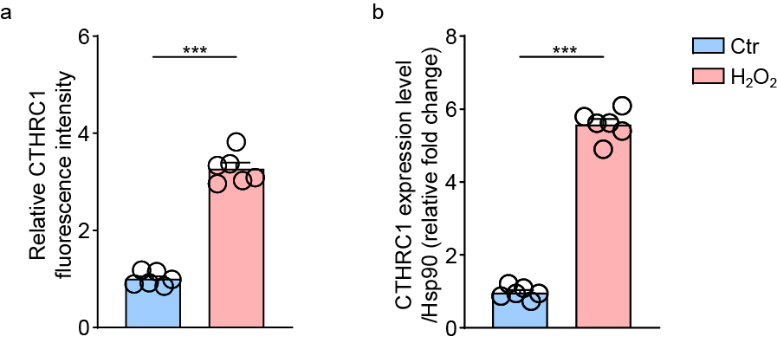


**Figure S7. a.** Quantification of immunofluorescence of TSPC stained with CTHRC1 antibody (n = 6). **b.** Quantification of western blot of CTHRC1 in different TSPC groups (n = 6). Data are presented as mean ± SEM. Unpaired t-test was used. ^***^*P* < 0.001.


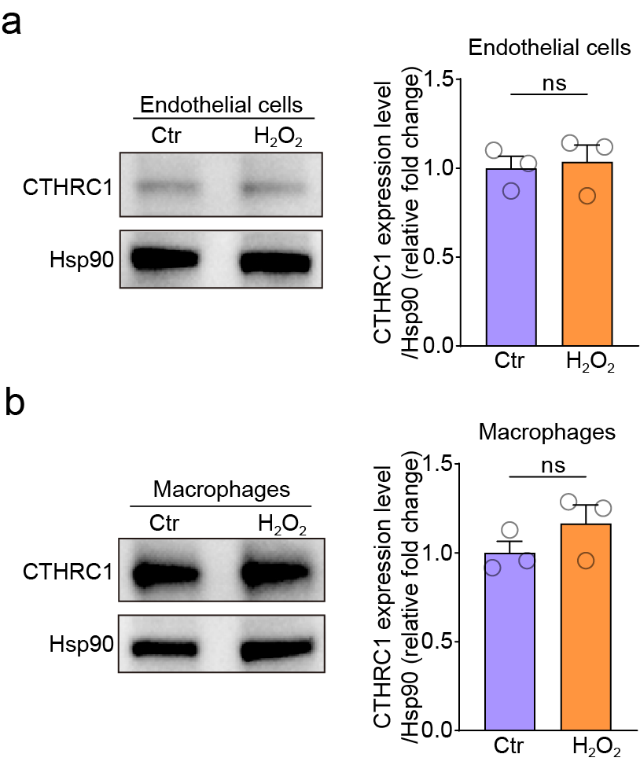


**Figure S8. a.** Representative western blot of CTHRC1 in endothelial cells with or without H_2_O_2_ and relative quantification (n = 3). **b.** Representative western blot of CTHRC1 in macrophages with or without H_2_O_2_ and relative quantification (n = 3). Data are presented as mean ± SEM. Unpaired t-test was used. ns, no significance.


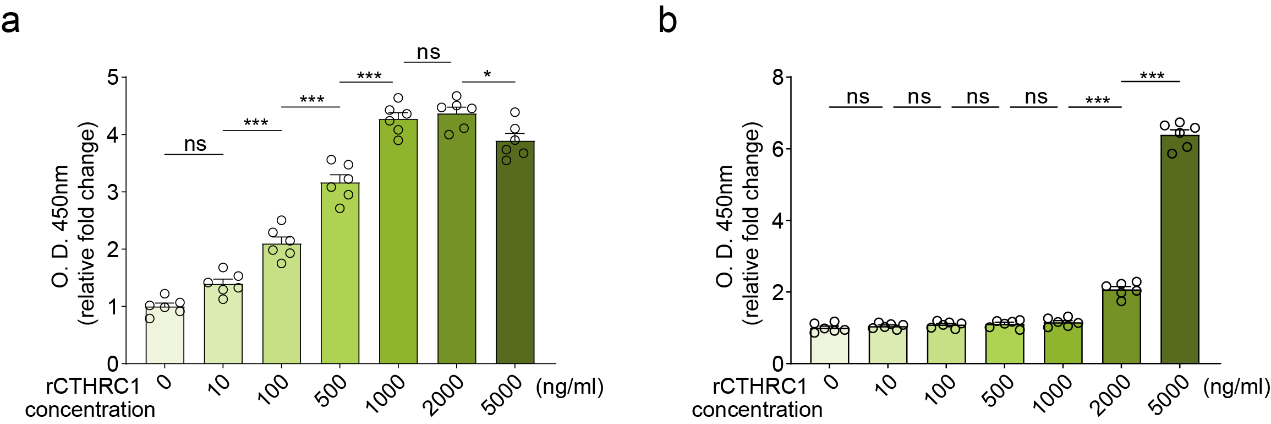


**Figure S9.** **a.** The cell viability was accessed in TSPC with different concentrations of rCTHRC1 (n = 6). **b.** The cytotoxic effect with different concentrations of rCTHRC1 in TSPC was measured using the LDH assay (n = 6). Data are presented as mean ± SEM. One-way ANOVA was used. ^*^*P* < 0.05. ^***^*P* < 0.001. ns, no significance.


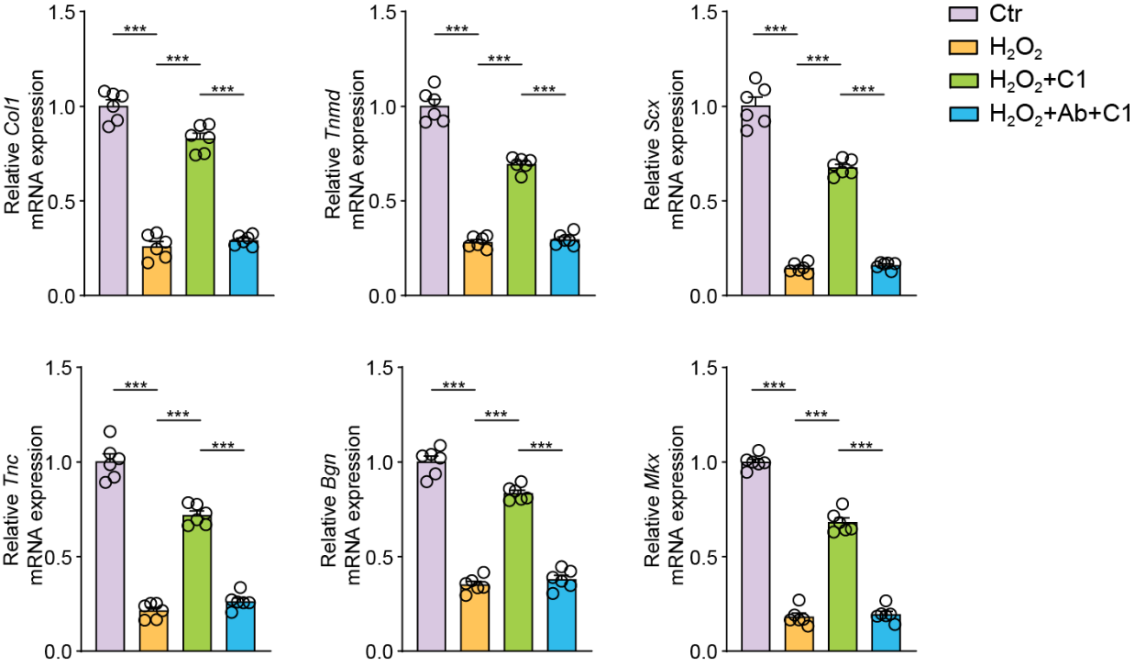


**Figure S10.** Relative fold change of indicated mRNA expression level in TSPC with different disposes (n = 6). Data are presented as mean ± SEM. One-way ANOVA was used. ^***^*P* < 0.001. C1: CTHRC1; Ab: anti-CTHRC1 antibody.


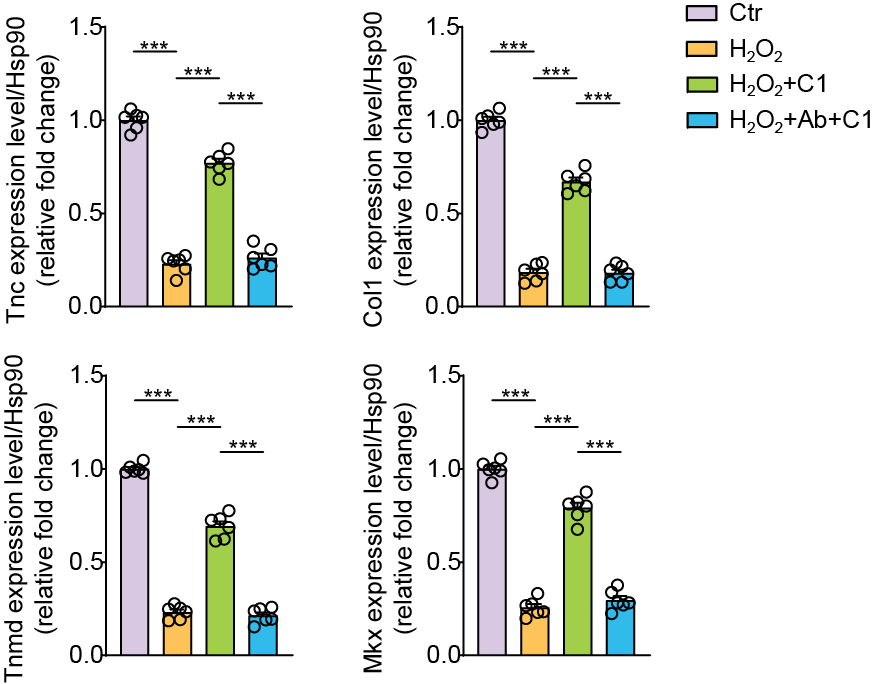


**Figure S11.** Quantification of western blot of indicated proteins in TSPC (n = 6). Data are presented as mean ± SEM. One-way ANOVA was used. ^***^*P* < 0.001. TSPC: tendon stem/progenitor cell; C1: CTHRC1; Ab: anti-CTHRC1 antibody.


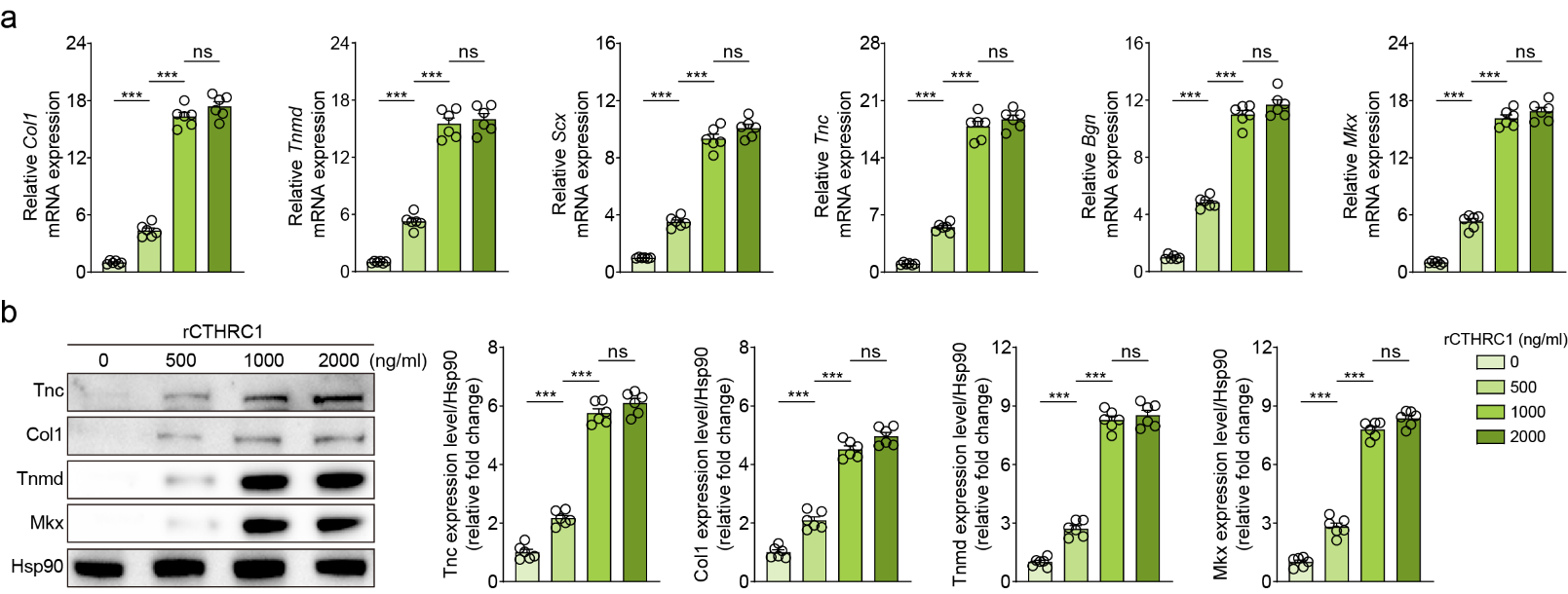


**Figure S12. TSPC** **tenogenic** **potentials in vitro promoted by rCTHRC1 in different concentrations.** **a.** Relative fold change of indicated mRNA expression level in TSPC with different concentrations of rCTHRC1 (n = 6). **b.** Representative western blot of indicated proteins in TSPC with different concentrations of rCTHRC1 and quantification (n = 6). Data are presented as mean ± SEM. One-way ANOVA was used. ^***^*P* < 0.001. ns, no significance.


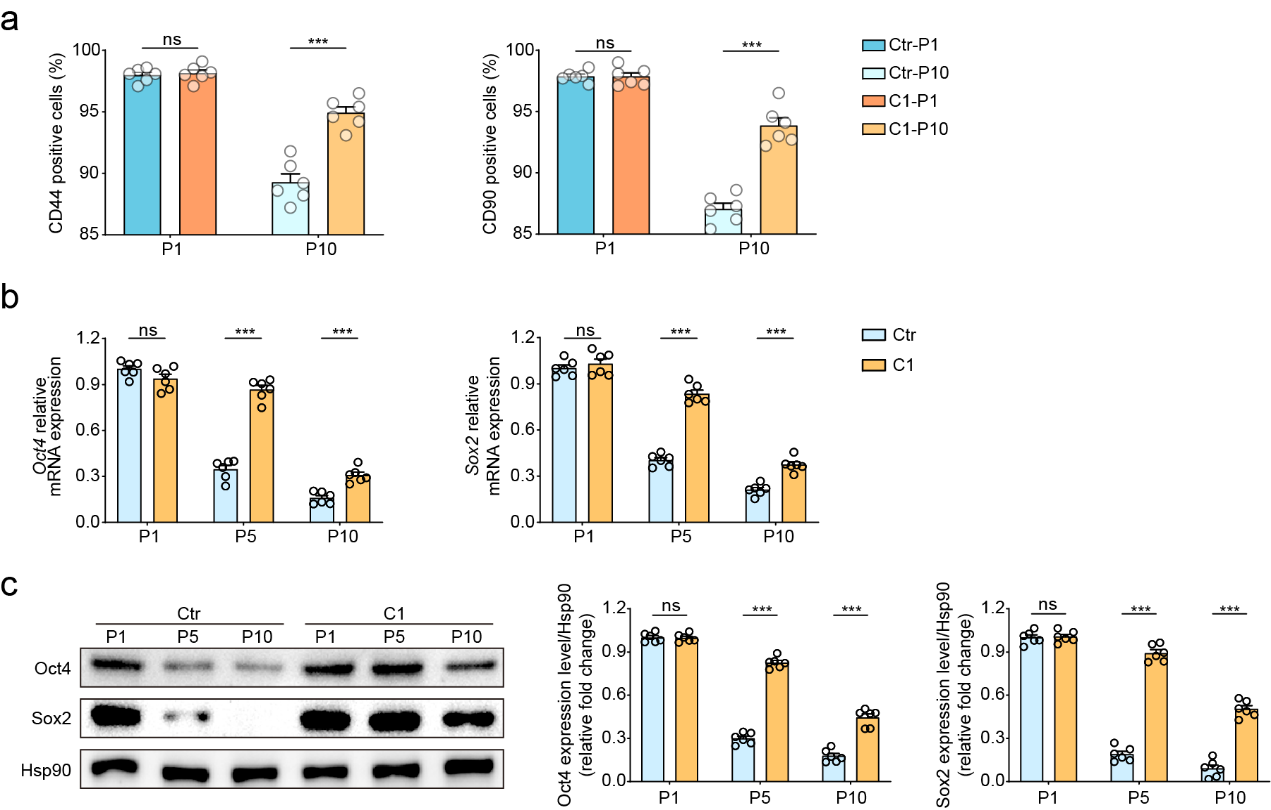


**Figure S13. TSPC stemness maintained in passage in vitro by rCTHRC1. a.** Quantification of flow cytometric analysis of TSPC with passages (n = 6). **b.** Relative fold change of Oct4 and Sox2 mRNA expression in TSPC (n = 6). **c.** Representative western blot of Oct4 and Sox2 in TSPC, as well as quantification (n = 6). Data are presented as mean ± SEM. Two-way ANOVA was used. ^***^*P* < 0.001. ns, no significance. TSPC: tendon stem/progenitor cell; C1: CTHRC1; P: passage.


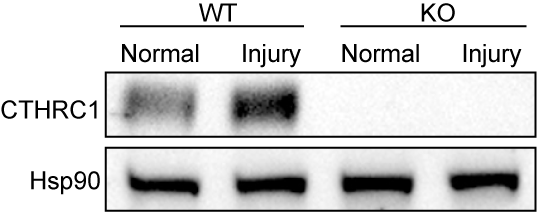


**Figure S14.** Validation of CTHRC1 knockout in mice’s Achilles tendon by western blot. WT: wild-type; KO: CTHRC1 knockout.


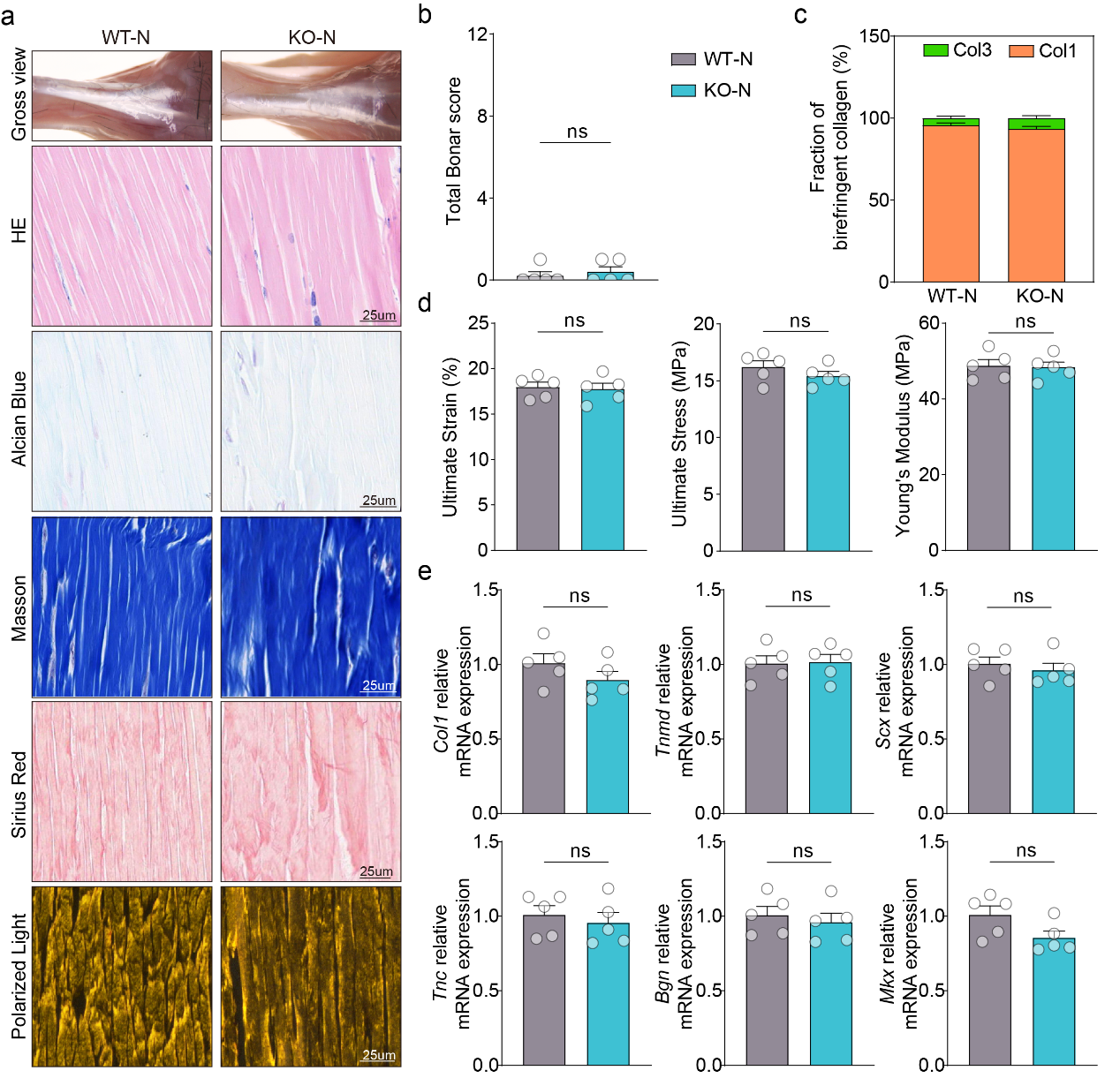


**Figure S15. Comparisons of Achilles tendon between normal wild-type mice (WT-N) and normal CTHRC1 knockout mice (KO-N) showed** **no significant difference. a.** Representative images of gross view, hematoxylin & eosin (HE), Alcian Blue, Masson, Sirius Red staining, and Sirius Red staining using polarized light microscopy. **b.** Assessment of Achilles tendon using the total Bonar score (n = 5). **c.** Fraction of birefringent collagen in Sirius Red staining imaged under polarized light (n = 5). **d.** Quantification of ultimate strain, ultimate stress, and Young’s modulus of mice’s Achilles tendon (n = 5). **e.** Relative fold change of indicated mRNA expression level in mice’s Achilles tendon (n = 5). Data are presented as mean ± SEM. **b**, by Mann-Whitney test; **d** and **e**, by unpaired t-test. ns, no significance. WT: wild-type; KO: CTHRC1 knockout; N: normal.


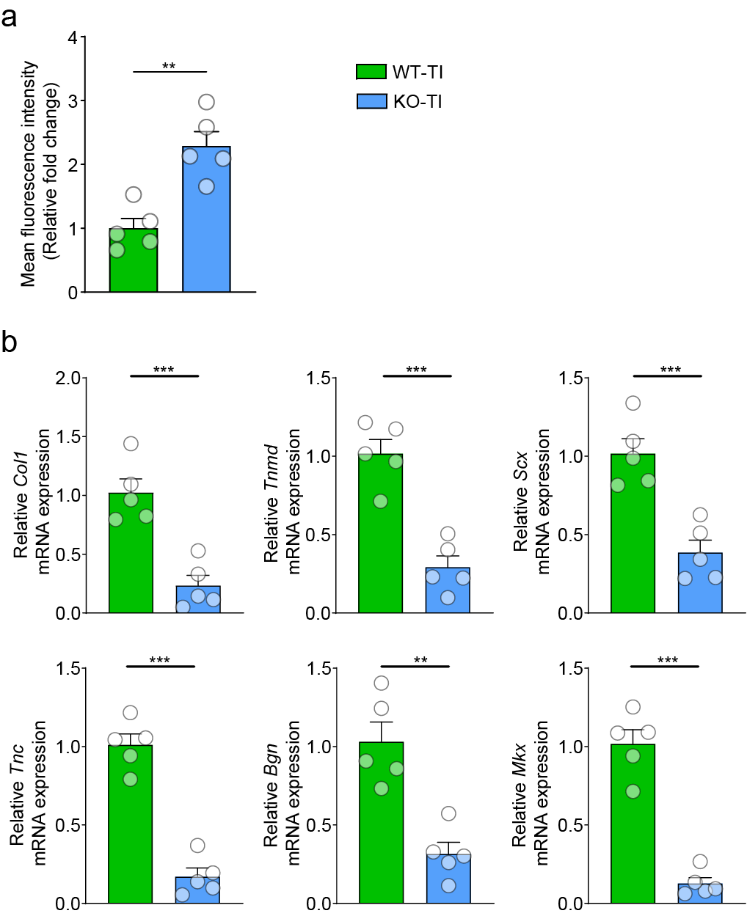


**Figure S16. a.** Quantification of mean fluorescence intensity (n = 5). **b.** Relative fold change of indicated mRNA expression level in mice’s Achilles tendon (n = 5). Data are presented as mean ± SEM. Unpaired t-test was used. ^**^*P* < 0.01; ^***^*P* < 0.001. WT: wild-type; KO: CTHRC1 knockout; TI: tendon injury.


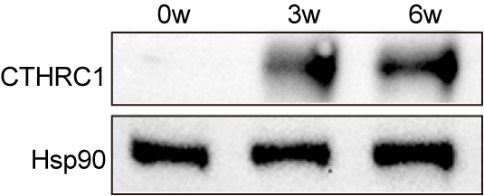


**Figure S17.** Validation of adeno-associated virus overexpressing CTHRC1 (AAV-C1) in KO mice’s Achilles tendon by western blot.


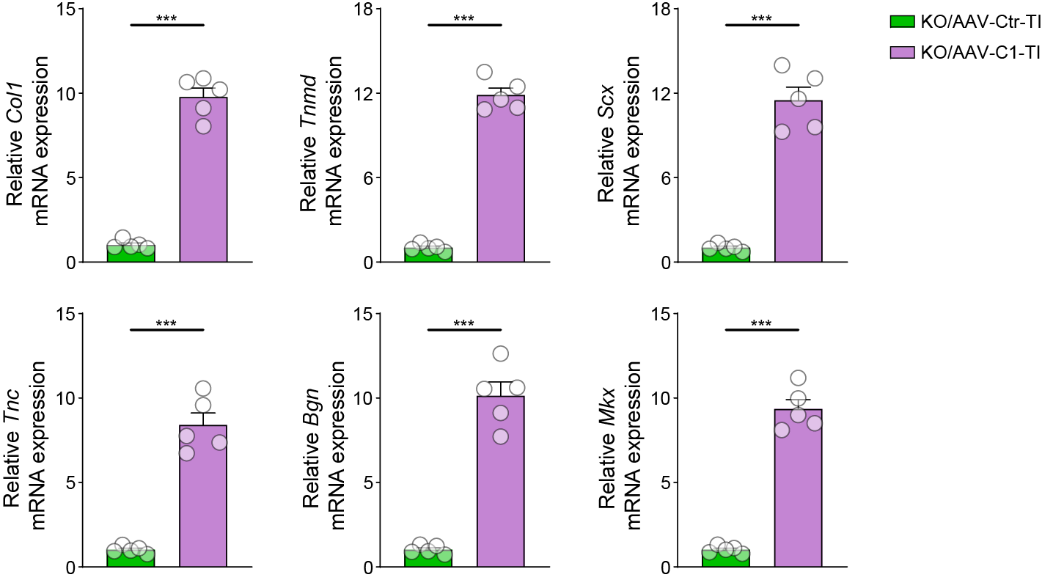


**Figure S18.** Relative fold change of indicated mRNA expression level in mice’s Achilles tendon (n = 5). Data are presented as mean ± SEM. Unpaired t-test was used. ^***^*P* < 0.001. KO: CTHRC1 knockout; AAV-C1: adeno-associated virus overexpressing CTHRC1; AAV-Ctr: empty AAV vector; TI: tendon injury.


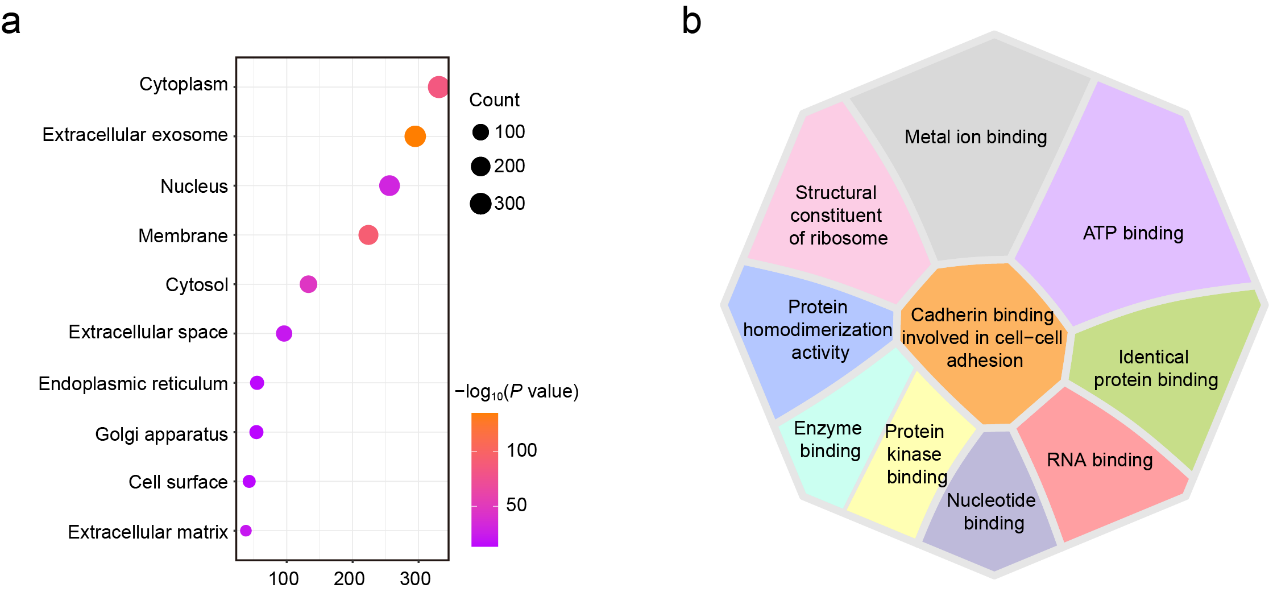


**Figure S19.** **a.** Bubble diagram of GO cell component. **b.** Enriched GO terms by molecular function. The area represents enrichment.


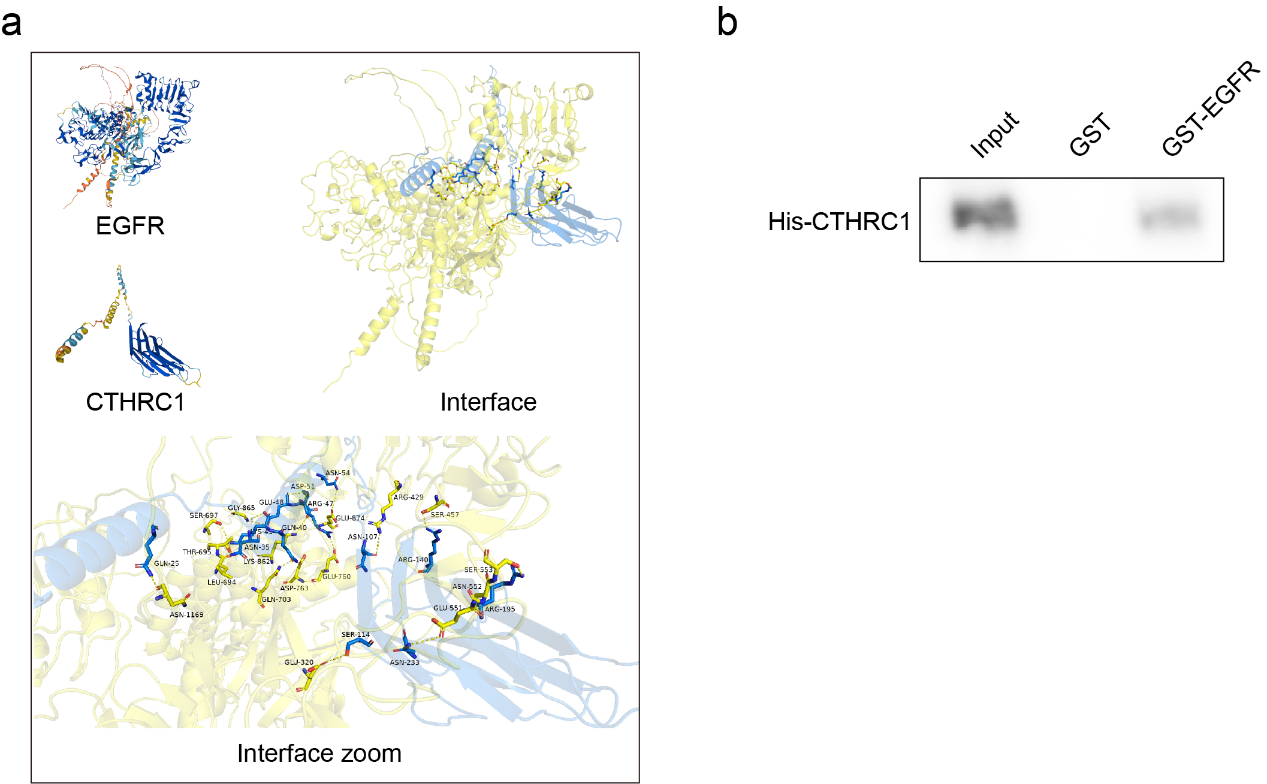


**Figure S20. a.** The structure of CTHRC1 and EGFR with their molecular docking model. **b.** GST pull-down assay showing that CTHRC1 directly interacted with EGFR.


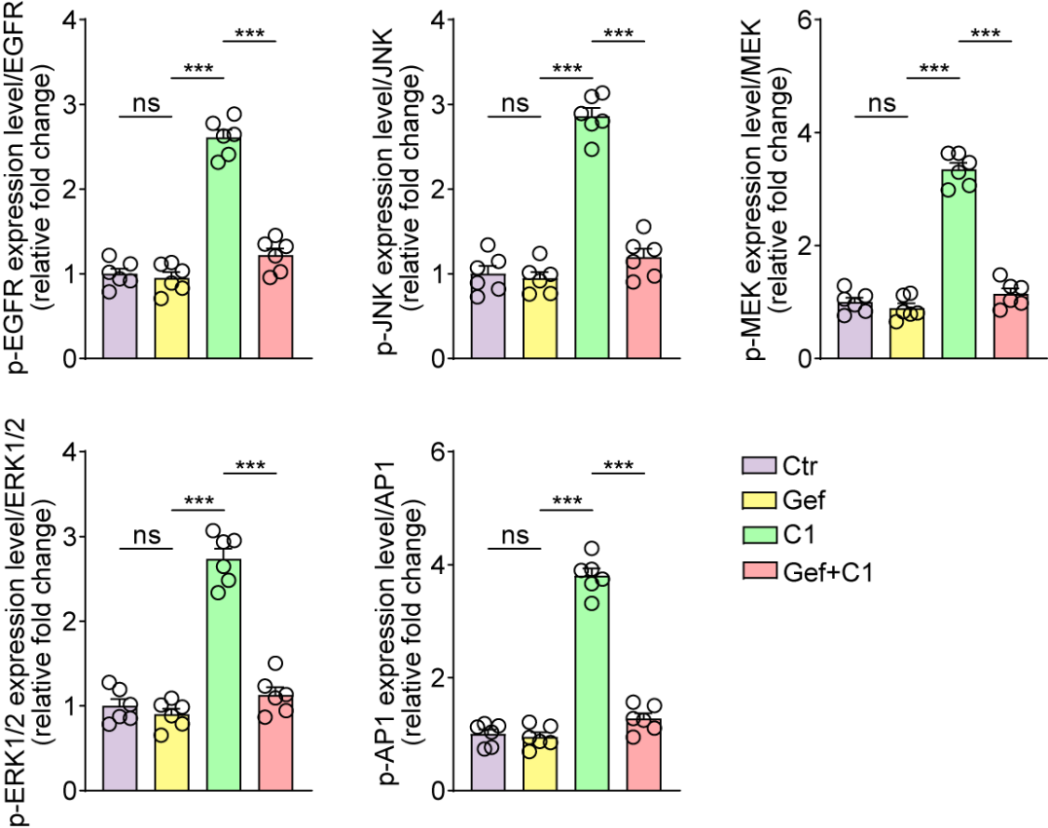


**Figure S21.** Quantification of western blot of indicated proteins in TSPC with different disposes (n = 6). Data are presented as mean ± SEM. One-way ANOVA was used. ^***^*P* < 0.001. ns, no significance. Gef: gefitinib; C1: CTHRC1.


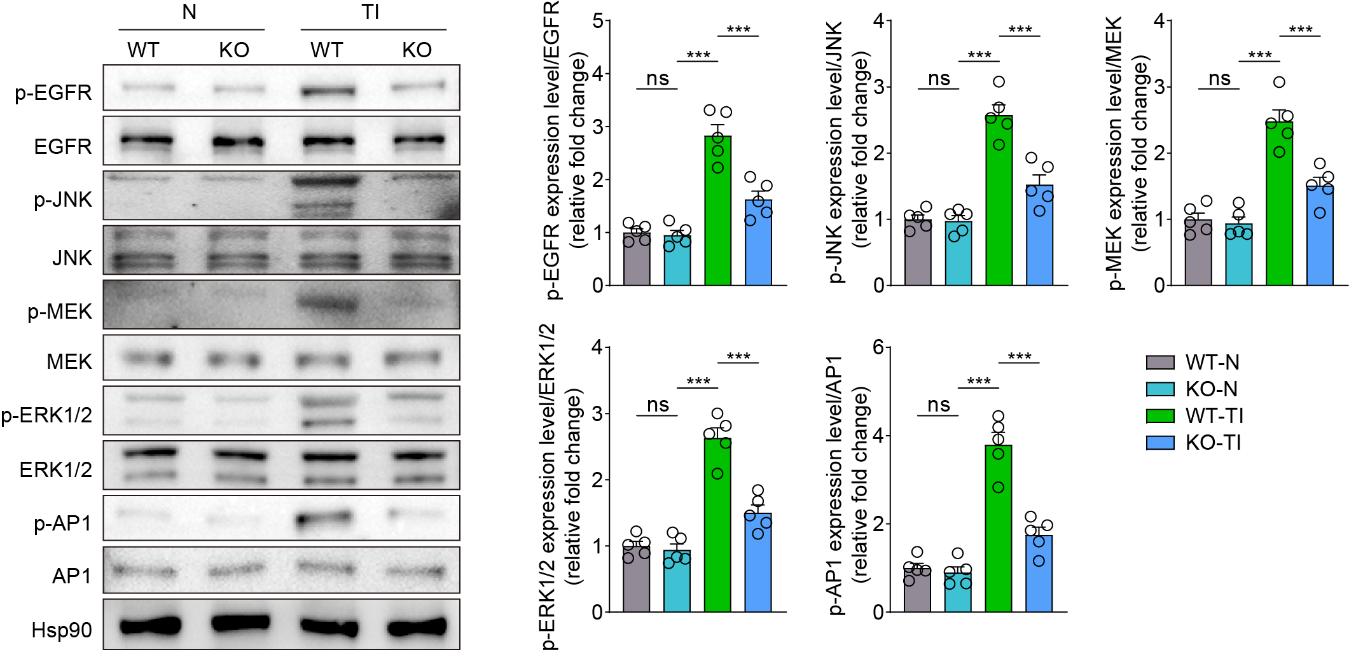


**Figure S22.** Representative western blot of indicated proteins in mice’s Achilles tendon with different disposes and quantification (n = 5). Data are presented as mean ± SEM. One-way ANOVA was used. ^***^*P* < 0.001. ns, no significance. WT: wild-type; KO: CTHRC1 knockout; N: normal; TI: tendon injury.


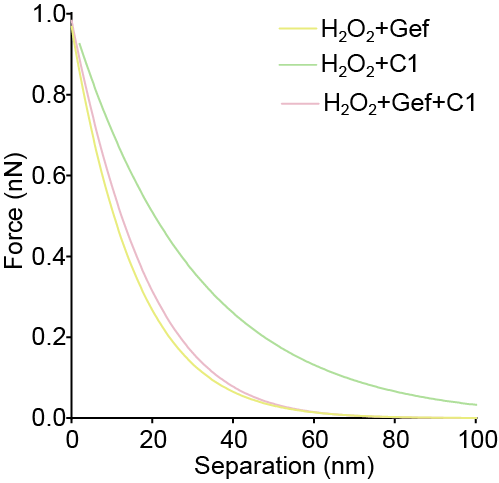


**Figure S23.** Representative AFM curves of TSPC. Gef: gefitinib; C1: CTHRC1.


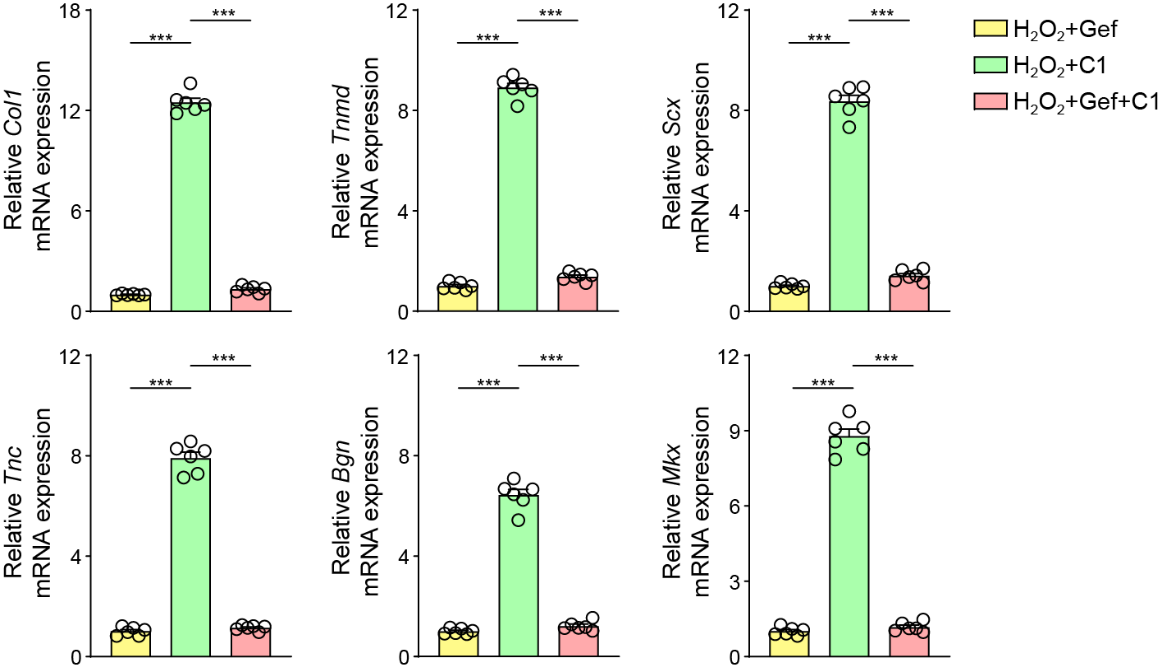


**Figure S24.** Relative fold change of indicated mRNA expression level in TSPC with different disposes (n = 6). Data are presented as mean ± SEM. One-way ANOVA was used. ^***^*P* < 0.001. Gef: gefitinib; C1: CTHRC1.


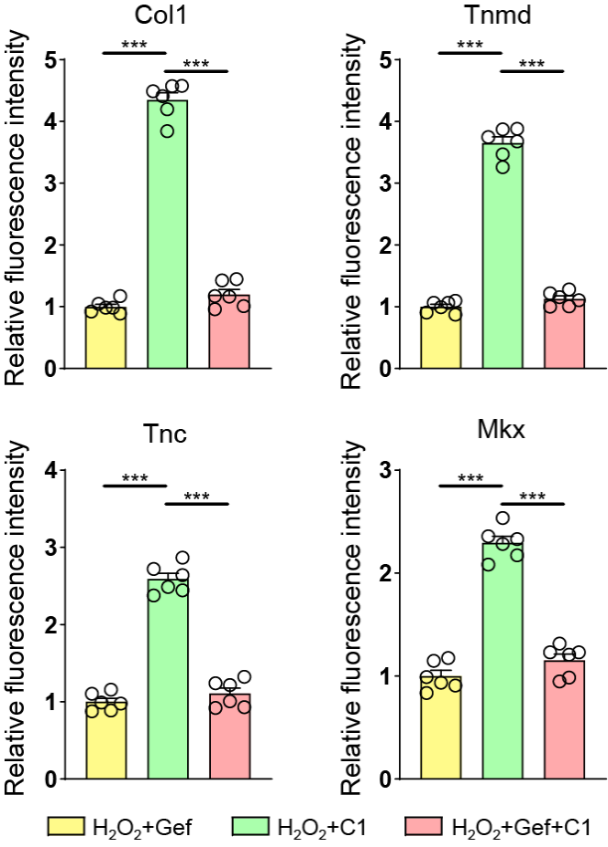


**Figure S25.** Quantification of indicated fluorescence intensity in TSPC with different disposes (n = 6). Data are presented as mean ± SEM. One-way ANOVA was used. ^***^*P* < 0.001. Gef: gefitinib; C1: CTHRC1.


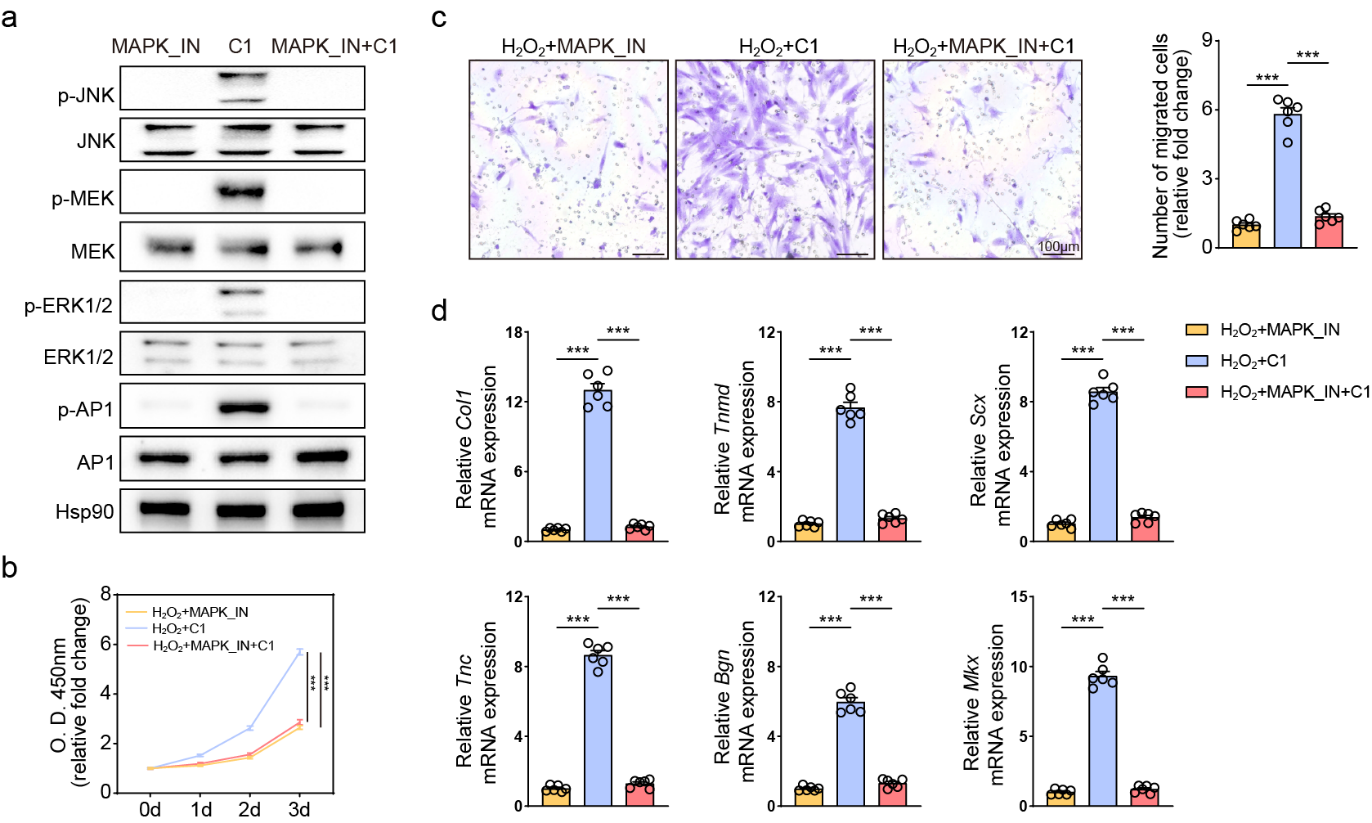


**Figure S26. MAPK** **inhibitor reverses the effects of CTHRC1 on the proliferation, migration, and tenogenic differentiation of TSPC in vitro. a.** Western blot images of indicated proteins in TSPC. **b.** TSPC proliferation curves at indicated time (n = 6). **c.** Representative images of transwell assay at 24 hours and quantification (n = 6). **d.** Relative fold change of indicated mRNA expression level in TSPC (n = 6). Data are presented as mean ± SEM. **b**, by two-way ANOVA; **c** and **d**, by one-way ANOVA. ^***^*P* < 0.001. C1: CTHRC1; MAPK_IN: MAPK inhibitor.


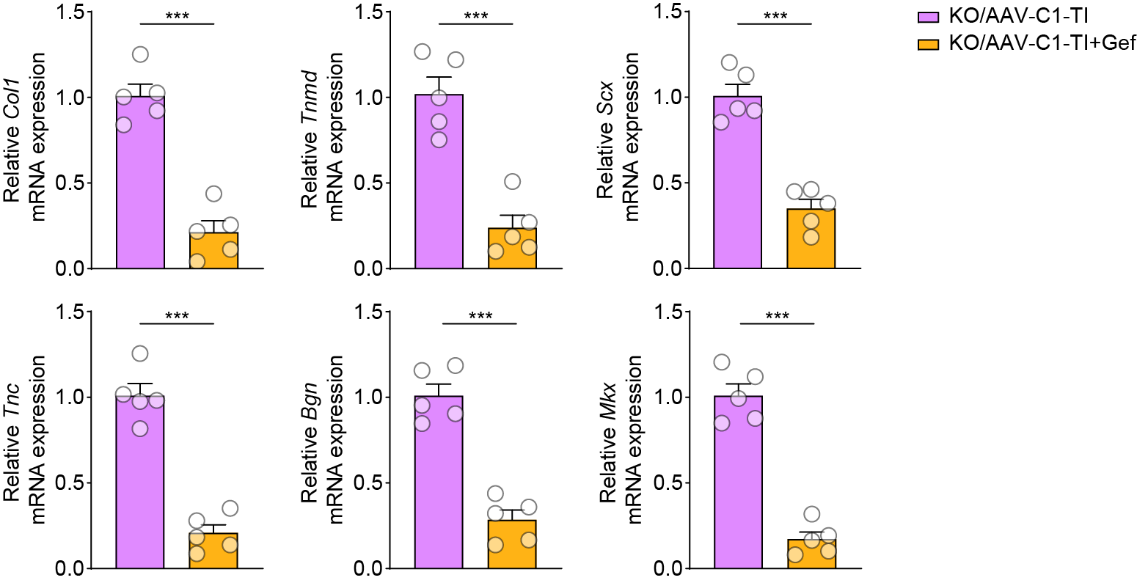


**Figure S27.** Relative fold change of indicated mRNA expression level in mice’s Achilles tendon (n = 5). Data are presented as mean ± SEM. Unpaired t-test was used. ^***^*P* < 0.001. KO: CTHRC1 knockout; AAV-C1: adeno-associated virus overexpressing CTHRC1; TI: tendon injury; Gef: gefitinib.


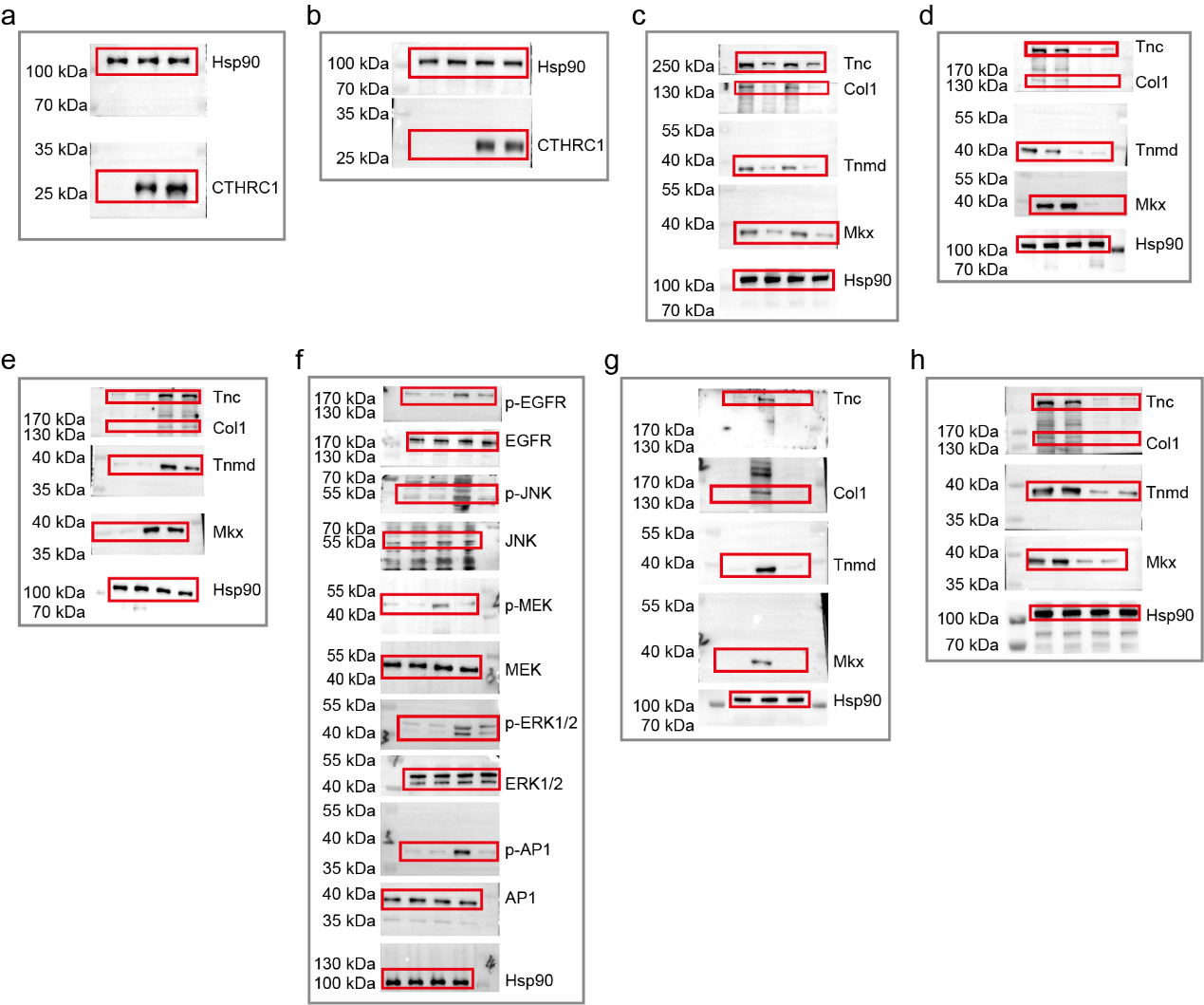


**Figure S28.** The original western blot images presented respectively. **a.** The original western blot images of Figure 1h. **b.** The original western blot images of Figure 1n. **c.** The original western blot images of Figure 2i. **d.** The original western blot images of Figure 3l. **e.** The original western blot images of Figure 4l. **f.** The original western blot images of Figure 5k. **g.** The original western blot images of Figure 6h. **h.** The original western blot images of Figure 7l.

**Table S1**. Hydrogen bonds

| CTHRC1 | Distance. [Å] | EGFR |
| --- | --- | --- |
| C: GLN 25 [NE2] | 2.39 | A: ASN 1169 [0] |
| C: ASN 35 [ND2] | 3.65 | A: LEU 694 [0] |
| C: GLN 40 [NE2] | 3.16 | A: ASP 763 [OD1] |
| C: LYS 41 [NZ] | 3.36 | A: SER 697 [OG] |
| C: LYS 41 [NZ] | 3.66 | A: THR 695 [0] |
| C: ARG 47 [NH2] | 2.54 | A: GLU 760 [OE1] |
| C: ASN 54 [ND2] | 3.76 | A: GLU 874 [OE1] |
| C: SER 114 [OG] | 3.8 | A: GLU 320 [OE1] |
| C: ARG 140 [NH1] | 2.38 | A: SER 457 [OG] |
| C: ARG 195 [N] | 2.3 | A: ASN 552 [OD1] |
| C: ARG 195 [NH2] | 3.73 | A: SER 553 [OG] |
| C: ASN 233 [ND2] | 3.83 | A: GLU 551 [OE1] |
| C: GLN 40 [OE1] | 2.23 | A: GLN 703 [NE2] |
| C: GLU 48 [OE1] | 3.54 | A: GLY 865 [N] |
| C: ASP 51 [OD2] | 3.84 | A: LYS 862 [NZ] |
| C: ASN 107 [0] | 3.81 | A: ARG 429 [NH2] |
